# New Capability in Autonomous Ocean Carbon Observations Using the Autosub Long-Range AUV Equipped with Novel pH and Total Alkalinity Sensors

**DOI:** 10.1021/acs.est.4c10139

**Published:** 2025-04-01

**Authors:** Emily M. Hammermeister, Stathys Papadimitriou, Martin Arundell, Jake Ludgate, Allison Schaap, Matthew C. Mowlem, Sara E. Fowell, Edward Chaney, Socratis Loucaides

**Affiliations:** † School of Ocean and Earth Sciences, University of Southampton, SO17 1BJ Southampton, United Kingdom; ‡ National Oceanography Centre, European Way, SO14 3ZH Southampton, United Kingdom

**Keywords:** ocean carbon observations, marine carbonate system, oceanographic sensors, ocean acidification, autonomous observations, autonomous underwater vehicles

## Abstract

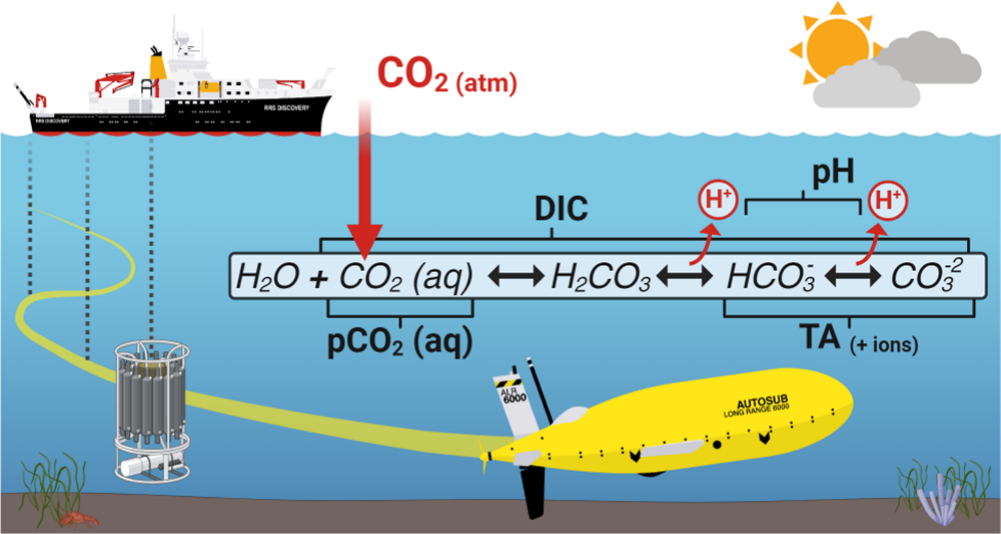

The development of marine autonomous platforms has improved our capability to gather ocean observations at fine spatial scales and high temporal frequency, which can be used to better measure, characterize, and model ocean carbon. As part of the OCEANIDS program, novel carbonate sensors were integrated into the Autosub Long-Range (ALR) autonomous underwater vehicle (AUV) and deployed in the Celtic Sea. Autonomous Lab-On-Chip (LOC) sensors measured pH and total alkalinity (TA) while onboard the ALR. Using interpolation, the ALR-sensor data set is compared against CTD co-samples. The average differences between the LOC sensor and co-sample pH range from −0.011 to −0.015. The TA sensor data agrees with co-samples within 1–2 μmol kg^–1^ on average. Biogeochemical water properties differing between CTD and ALR observations reveal correlations to carbonate parameter variations. The LOC sensors enabled the characterization of the marine carbonate system from autonomous subsurface measurements for the first time. Sensor pH and TA data were used to calculate dissolved inorganic carbon (DIC), partial pressure of CO_2_ (pCO_2_), and aragonite saturation state (Ω_Ar_) and are compared with CTD co-samples with mean residuals of 4–7 μmol kg^–1^, 10–17 μatm, and −0.03 to −0.06, respectively. Future perspectives on sensor deployment and analysis are discussed.

## Abstract 2

Advancements in autonomous technology for measuring ocean carbon provide new capabilities and insights for monitoring the ocean’s role in global carbon regulation.

## Introduction

It has long been established that carbon dioxide (CO_2_) levels in the atmosphere are rising. Since the start of the industrial era, total atmospheric CO_2_ concentration has increased by over 50%, with current anthropogenic emissions surpassing 11 Gt C yr^–1^.^1^ The ocean is a natural carbon sink that, in the past decade, has absorbed 2.9 ± 0.4 Gt C yr^–1^, equal to 26% of total anthropogenic CO_2_ emissions.^1,2^ As atmospheric CO_2_ dissolves into the ocean, it reacts with seawater to form carbonic acid, which dissociates into bicarbonate (HCO_3_^–^), carbonate (CO_3_^2–^), and hydrogen (H^+^) ions.^3^ The net addition of H^+^ acidifies the seawater (lowers the pH) in a process known as ocean acidification,^4^ and changes the speciation within the carbonate system.^5,6^ While atmospheric CO_2_ continues to rise and the ocean continues to take up more CO_2_, the ocean’s capacity to absorb surplus anthropogenic CO_2_, or buffer global climate change, has decreased.^7,8^ In fact, recent models suggest the ocean’s buffer capacity could decrease as much as 34% by 2100, likely accelerating ocean acidification.^9^

Ocean acidification and the changing ocean carbonate system affect the basis of the marine food web and marine biogeochemical cycles. It is not only a threat to marine health, but to human prosperity by threatening food and economic security.^10^ Over 40% of the growing human population lives in coastal regions, making our dependence on the ocean’s resources ever-increasing.^11^

Reflecting the urgency and importance of understanding the changes to the ocean carbon system, the Global Ocean Observing System (GOOS) has deemed inorganic carbon (ocean carbonate system) as an essential ocean variable (EOV) to measure. Ocean carbon observations are essential for assessments of the ocean carbon budget and quantification of fluxes which, through the ocean carbon value chain, are used to inform policymakers and stakeholders on managing emissions and climate change mitigation strategies.^12^ However, the quality of these assessments is a function of the quality and availability of carbon data observations that have come from ship-based programs (e.g., GO-SHIP, SOOP, etc.). While these programs have provided critical insights into the ocean carbon cycle, data availability is scarce in time and space, leading to large uncertainties and discrepancies between models and observations^13,14^ that hinder policymaking progress and climate resolution.

Although offering the highest quality observations required to track climate-scale changes in the ocean’s carbon system, traditional ship-based observing strategies have several limitations including high operating costs, long transit times, and practical seasonal biases—especially in polar regions.^15^ Ship-based observations often fail to capture interannual variability and dynamic spatiotemporal variability in coastal oceans. Additionally, the carbon footprint of ship-based operations is facing increasing scrutiny as the world strives to achieve net-zero carbon emissions (e.g., Future Marine Research Infrastructure (https://fmri.ac.uk). Efficient and sustainable ocean observing strategies are therefore needed to increase measurement resolution in space and time, complementing ship-based methods in an effort to decarbonize marine research and meet current scientific and societal needs.

The emergence and expansion of autonomy in ocean observations, specifically platforms such as profiling floats, underwater gliders, and surface vehicles equipped with scientific sensors, offer a scalable, sustainable, and complementary solution to current observational needs. Even so, the lack of autonomous sensors for direct characterization of the ocean carbonate system remains the limiting factor to wide-scale and high-resolution ocean carbon observations.

To quantify and characterize the marine carbonate system, there are four measurable key variables to consider: Dissolved Inorganic Carbon (DIC), Total Alkalinity (TA), pH, and partial pressure of CO_2_ (pCO_2_).^16^ The carbonate system can be constrained by a system of stoichiometric equations so that any pair of these four parameters can be used (alongside salinity, temperature, and pressure) to calculate the remaining two.^17^ Currently, only sensors measuring pH and pCO_2_ are available commercially and are capable of autonomous observations. However, because of the covariance of these two parameters in the environment, their choice as input parameters is less desirable since it leads to large uncertainties in the characterization of the carbonate system.^18,19^ In the absence of commercial, integrable sensors capable of directly measuring TA or DIC in situ, characterizations of the carbonate system based on autonomous platform observations (e.g., BGC-Argo and SOCCOM programs) rely on TA estimated from empirical algorithms using salinity, oxygen, and nutrients as input parameters.^20,21^ Although this approach provides a good alternative to direct observations, its applicability and reliability vary.

There has been recent work to integrate carbonate sensors onto autonomous platforms, including Autonomous Surface Vehicles (ASVs) and gliders. For example, the Saildrone and Wave Glider ASVs have been equipped with pCO_2_ (ASVCO_2_) systems,^22,23^ and proved to be a valuable tool for CO_2_ flux quantification, especially in hard-to-reach environments. Additionally, the first-ever integration of a Lab-On-Chip (LOC) pH sensor onto a glider, by Possenti et al.,^24^ provided valuable insights into biogeochemical interactions and processes in the North Sea. Similar to BGC-Argo floats, Possenti was able to derive carbonate variables by using autonomous pH data paired with salinity-derived TA estimations. Despite these advancements, autonomous instrumentation falls short of fully matching the comprehensive carbonate data collection capabilities of traditional ship-based methods.

In this article, we introduce novel state-of-the-art autonomous observing technologies capable, for the first time, of direct seawater–carbonate system characterization along predefined oceanographic transects. The system comprises a long-range Autonomous Underwater Vehicle (AUV) newly loaded with LOC sensors for the measurement of seawater pH, TA, and DIC. We evaluate the performance of these new technologies—including the quality of observations and ability to constrain the carbonate system—relative to the traditional ship-based approach.

## Methods

### Study Site

The study site was located in the Celtic Sea, between the Celtic Shelf and Deep Celtic Basin in ocean waters ranging from 100 to 3000 m deep. This region was chosen due to its proximity to the UK and the presence of biogeochemical gradients across the continental shelf break. The study took place between March 19–30, 2022 supported by the Royal Research Ship, *RRS Discovery*, during expedition DY149.

### Autonomous Platform and Sensors

The Autosub Long Range (ALR) is a family of large flight-style AUVs (3.6 m long, nominally weighing 750 kg) developed and operated by the National Oceanography Centre (NOC) with a depth rating of 1500 m (ALR1500) or 6000 m (ALR6000). The ALR-2 (ALR6000) was used for this deployment and is hereafter referred to as the ALR. The ALR has flooded payload bays that sit forward and aft and can be configured with a wide range of oceanographic sensors. Long endurance is achieved using lithium batteries combined with low transport costs from modest travel speeds, passive buoyancy control, and optimized power consumption of onboard systems.^25^ A propeller, magnetically coupled to an electric motor and gearbox enables speeds between 0.45 and 0.8 m s^–1^ through water. Large aerofoil section dive wings provide downward force and control surfaces at the aft that manage pitch and heading. Typical dive rates of 0.1–0.3 m s^–1^ are achieved with a downward pitch of 10–30°. The ALR navigates using Doppler Velocity Log (DVL) aided dead reckoning, achieving navigational accuracy <1% of the distance traveled when in range of the seabed.^25,26^

For this deployment, the ALR was fitted with three additional major sensor suites: carbonate chemistry LOC sensors (pH, TA, and DIC), nutrient LOC sensors (nitrate/nitrite, phosphate, silicate, and iron), and a single turnover active fluorescence (STAF) phytoplankton sensor. As a part of its standard payload, the ALR was equipped with a pumped CTD (SBE 52-MP) sensor, and for this work, an SBE 43F dissolved oxygen (DO) sensor was added to the CTD. All three carbonate LOC sensors are rated to 6000 m^27−29^; however, the maximum depth rating for this deployment was reduced from 6000 to 600 m due to the limited pressure rating of the STAF sensor. The hotel load (systems and science payload) in this configuration was 60 W giving an expected endurance of 10 days and a range of 550 km at a speed of 0.6 m s^–1^.

In this work, we focus on the CTD, DO, pH, TA, and DIC sensors and their capability within the autonomous system to characterize the ocean carbonate system. The pH LOC sensor determines pH on the total proton scale (pH*_T_*) photometrically using purified meta-Cresol Purple (mCP) as the indicator dye, with <0.001 precision, 0.003 ± 0.022 accuracy relative to validation seawater samples, and ±0.010 combined standard measurement uncertainty.^27^ The pH LOC sensor has been widely demonstrated in remote operated vehicles (ROVs), seabed landings, and gliders^24,30−32^ and is now commercially available (https://www.clearwatersensors.com/). The TA LOC sensor determinations are based on single-point acid titration to endpoint pH = 3.0–3.5 that is determined photometrically using (unpurified) Bromophenol Blue (BPB) as the indicator in the hydrochloric acid (HCl) titrant.^33^ The TA sensor has a precision and accuracy better than 5 μmol kg^–1^,^28^ and the TA measurement uncertainty is estimated to be ±7 μmol kg^–1^ in this deployment. Full details of the calibration and validation procedures of the pH and TA LOC sensors are outlined in previous studies.^27,28^ While the temperature and optical calibrations of these sensors are performed once postmanufacturing, validation is typically performed with standardized (e.g., ‘tris’ buffer, validation co-samples^27^) or certified materials pre-deployment, during deployment with onboard materials, and postdeployment (TA sensor only).

The DIC LOC sensor is based on the conductometric method in Hall and Aller,^34^ which involves extraction of DIC as CO_2_ from a seawater sample by acidification with 10% phosphoric acid, CO_2_ transfer into a 0.007 M sodium hydroxide (NaOH) solution across a gas permeable membrane, and determination of the conductivity change in the NaOH solution from its reaction with CO_2_ to CO_3_^2–^.^35^ The calibration procedure of the conductometric DIC LOC sensor is based on that outlined in Sayles and Eck.^35^ The DIC sensor used for this deployment was an early, now retired, prototype that featured an external detector with relatively high measurement uncertainty (estimated at ±38 μmol kg^–1^).^29^

**Figure 1:**
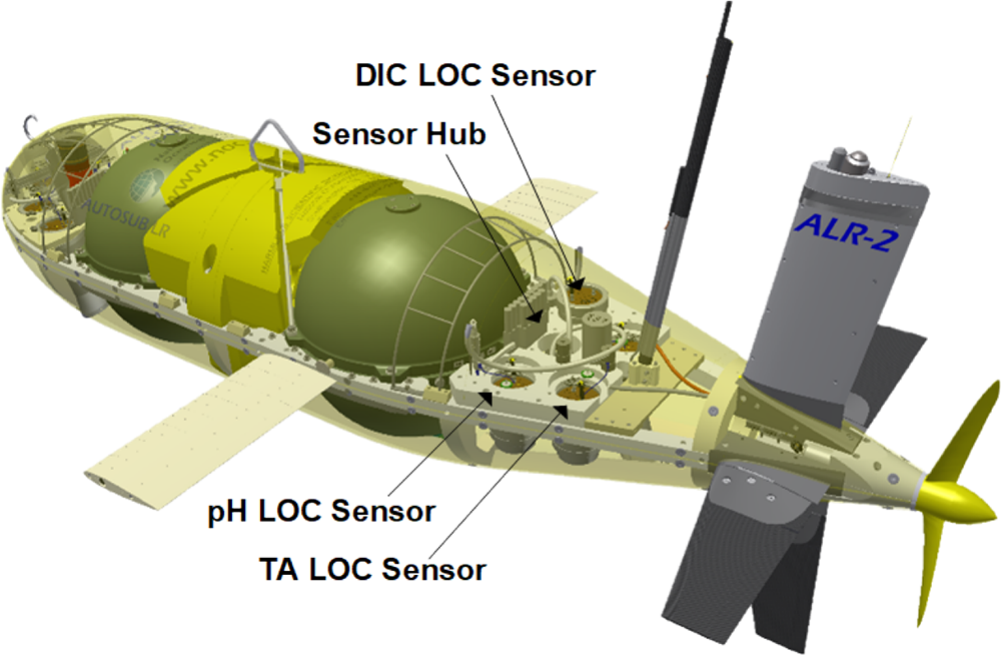
Diagram of the ALR with the carbonate LOC sensor and sensor hub integration in the aft payload bay. Reagent bags are not shown.

The carbonate sensors were integrated into the aft payload bay of the ALR (Figure 1). The integration of the carbonate sensors with the ALR was performed by using a communications sensor hub.

The sensor hub was developed specifically to simplify the integration of multiple sensors on autonomous platforms. It primarily operates as a port expander, providing a power and serial communications interface between a vehicle and multiple sensors. The hub is fully programmable, allowing it to perform any degree of protocol translation or other “smart” functions. In this application, the sensor hub managed the operation of the individual carbonate sensors and presented the ALR with an interface to a single “virtual carbonate sensor” that could start, stop, and poll for samples. Performance of the system was improved further by making real-time supplementary CTD and DO data available to the LOC sensors via a 1 Hz stream from the SBE 52-MP CTD sensor onboard the ALR which enabled real-time calculation of carbonate system parameters at in situ salinity (S), temperature (T), and pressure (P). The LOC and CTD-DO sensors sampled seawater from a shared flow-through system that pumped seawater from an intake tube outside of the ALR’s housing.

### Autonomous Missions

**Figure 2:**
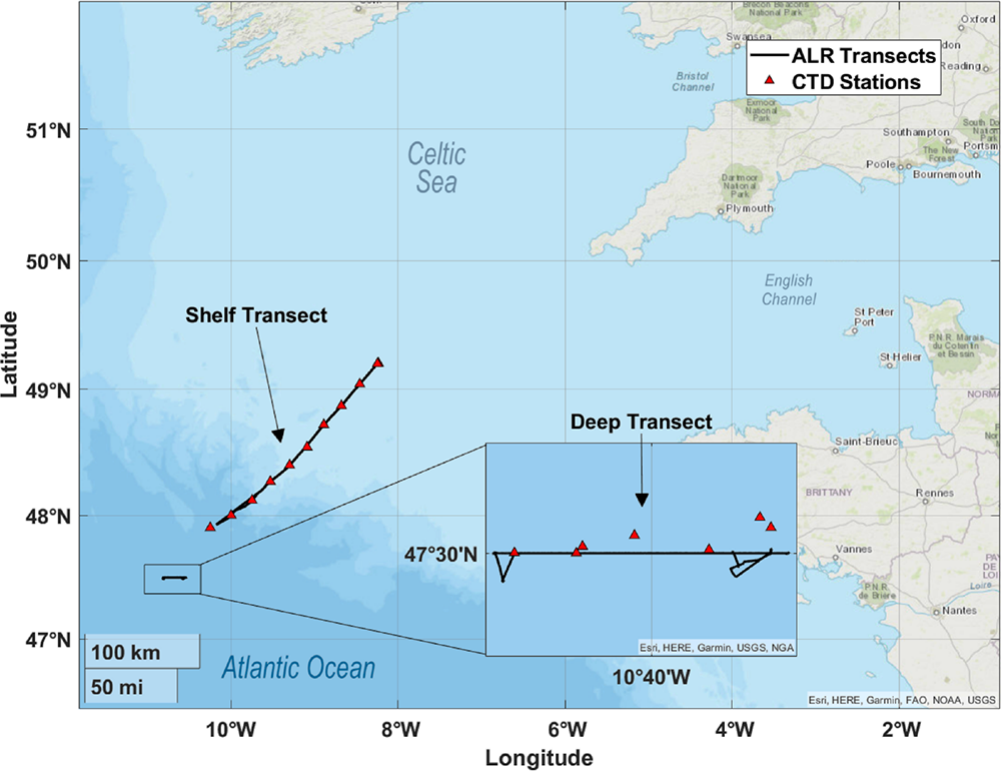
Sampling sites located Southeast off the coast of England in the Celtic Sea and off the Celtic Shelf in open ocean (Atlantic) waters.

Carbonate system observations were conducted through a series of ALR missions along two main transects: The Shelf Transect (ST) and the Deep Transect (DT) (Figure 2). During the ST missions, the ALR traveled in a southwesterly direction across the continental shelf over a period of 5 days (March 24–29, 2022).

**Figure 3:**
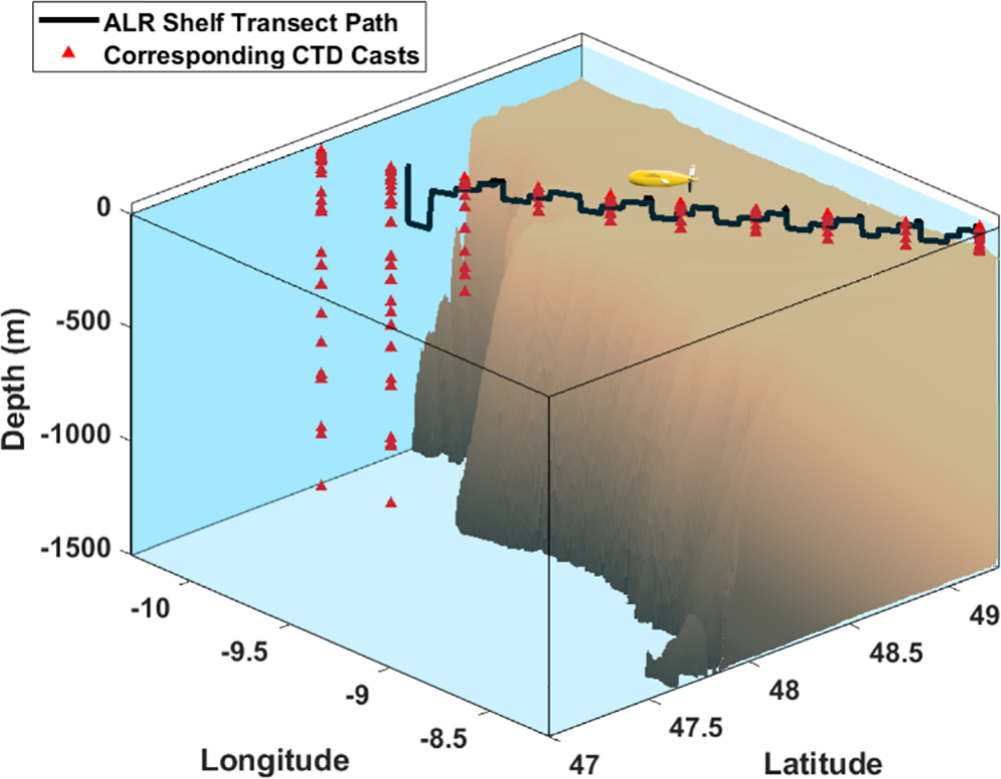
Visualization of ST ALR path (black line) and corresponding CTD casts (red triangles). ALR vehicle transit across the continental shelf edge at various depths in a diagonal trajectory with respect to latitude and longitude (denoted here in decimal degrees).

**Figure 4:**
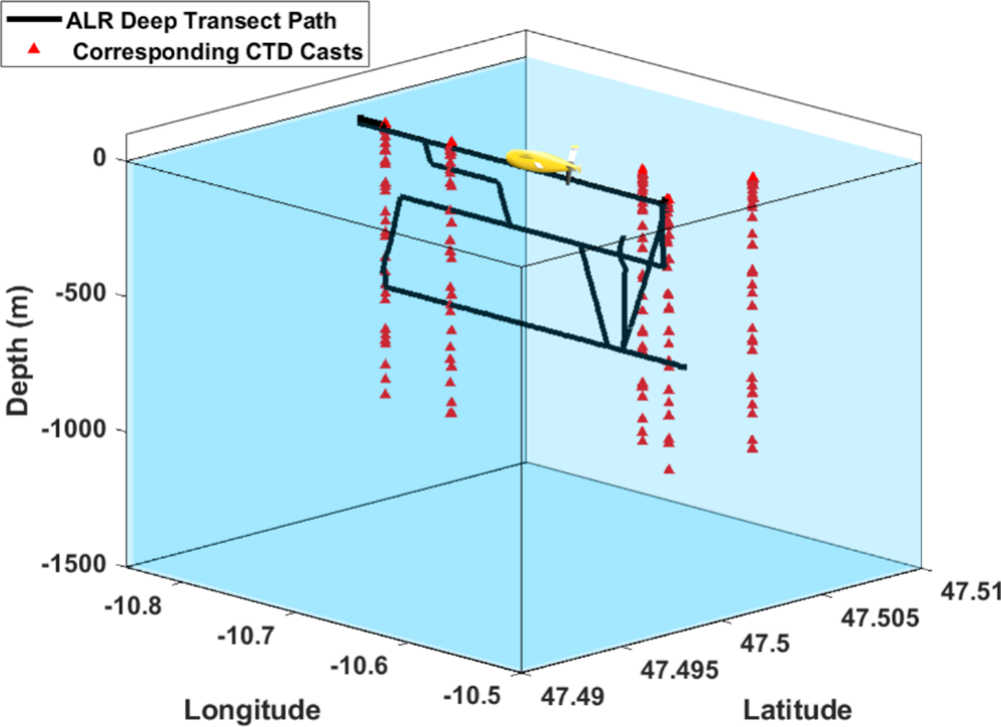
Visualization of DT ALR path (black line) and corresponding CTD casts (red triangles). Latitude and longitude are denoted here in decimal degrees. ALR vehicle transit path is along 47.5 N at various depths. Seafloor is at 3500 m.

The ALR followed a “staircase” survey pattern, reaching depths up to 250 m along a total distance of over 200 km (Figure 3). The DT mission took place just off the continental shelf over a period of 3 days (March 20–22, 2022). The ALR traveled along three 25 km stacked transects at 20, 250, and 600 m depth (Figure 4).

### Autonomous Data Processing

The carbonate LOC sensors were switched on by the ALR at the start of each dive sequence and measured at each of their maximum sampling frequencies, as outlined in Table 1. Each LOC sensor measurements were time-matched with the CTD-DO measurements by the hub and compiled within a single data file. The in situ S, T, and P were used to determine pH_T_ and TA as described in previous reports.^27,28^

**Table 1:** Sampling Rates of Sensors Onboard the ALR^a^

sensor on ALR	sampling rate
pH lab-on-chip (NOC)	1 measurement per 7.5 min
TA lab-on-chip (NOC)	1 measurement per 10 min
	RMs every 3 measurements*
DIC lab-on-chip (NOC)	1 measurement per 15 min
	RM every 12 measurements*
SBE 52 CTD (Sea-Bird Scientific)	1 Hz (1 measurement/second)
SBE 43F dissolved oxygen (Sea-Bird Scientific)	1 Hz (1 measurement/second)

a The LOC sensors were powered off after every mission when the ALR was at the surface trasmitting data and then were powered back on at the beginning of the next mission. The LOC sensors were set to measure at their highest possible frequency to ensure maximum data collection and prove ability rather than achieve measurement synchronization. *During deployment, onboard Reference Materials (RMs) were analyzed periodically for both the TA and DIC sensor.

The use of “CO2-in-seawater Reference Materials” (RMs), certified for TA and DIC, is a standard practice in marine carbonate system measurements. These RMs are also reliable tools for field sensor calibration and verification because they are stable for long periods of time and unaffected by changes in temperature and pressure.^36,37^ The TA sensor seawater measurements were determined relative to data collected periodically during deployment from the onboard RM and in-house prepared standard (seawater that has been filtered, poisoned with mercuric chloride, and standardized against RM and certified titrant, both from Scripps Institution of Oceanography, USA), as described in Schaap et al.^28^ The TA LOC sensor carried two onboard RMs, RM1 (certified, Scripps Batch 189) with TA = 2205.26 μmol kg^–1^ and RM2 (in-house standardized seawater with TA = 2340.8 μmol kg^–1^, which were each measured once every three external seawater measurements (Table 1). The DIC sensor used one onboard RM (certified, Scripps Batch 193) with DIC = 2048.36 μmol kg^–1^, which was measured in triplicate for every 12 external seawater measurements during deployment (Table 1).

### Discrete Bottled Co-samples

Discrete seawater co-samples were collected during ALR missions (Figures 2–4) using the ship’s CTD rosette sampler, equipped with 24 Niskin bottles (20 L each), a Sea-Bird SBE 911 plus CTD, a Sea-Bird SBE 43 dissolved oxygen sensor, and an Aquatracka MKIII fluorometer (Chelsea Technologies Group). For each CTD cast, seawater was collected from various depths, including the depth of the ALR track. Sample collection, preservation, and storage for carbonate analysis were conducted according to standard procedures described in Dickson et al.^38^ The bottled co-samples were analyzed in the laboratory, with a subset (*n* = 47) analyzed for pH_T_, DIC, and TA, at NOC and the remaining samples (*n* = 109) analyzed for DIC and TA at the Bermuda Institute in Ocean Science (BIOS). All discrete carbonate samples were analyzed within seven months of collection. Seawater pH_T_ (NOC) was determined on a Cary 60 UV–vis (Agilent Technologies) spectrophotometer using a purified mCP indicator at 20 °C,^38^ with an estimated uncertainty of 0.005^27^ (SI. Table 1). The reported in situ pH_T_ for bottled co-samples that were analyzed at NOC was computed from the laboratory-measured pH_T_, measurement temperature, DIC, and nutrient concentrations at in situ S, T, P using CO2SYS (see Carbonate system calculations section below). The in situ pH_T_ reported here for the remainder of bottled co-samples was computed from the DIC and TA measured at BIOS at in situ S, T, and P using CO2SYS. Seawater DIC (NOC) was determined by infrared (IR) gas analysis following acidification with 10% phosphoric acid and stripping of the generated CO_2_ with pure nitrogen gas on an AIRICA DIC Analyzer (Marianda, Kiel, Germany) coupled with a LICOR 840A IR CO_2_/H_2_O Analyzer.^39−41^ The analytical system was calibrated daily with RMs (Scripps Institution of Oceanography, USA). The DIC concentration was determined from two repeat measurements from the same discrete sample bottle, each measurement consisting of integrated CO_2_ peaks from four repeat injections of 1.2 mL of sample each, with a precision better than 6 μmol kg^–1^, and an average precision of 3 μmol kg^–1^ (1σ = 2 μmol kg^–1^). The same DIC determination method was used at BIOS, coupled with CO_2_ determination by coulometric titration on a VINDTA 3C (Versatile INstrument for the Determination of Total Alkalinity; Marianda, Kiel) with an accuracy and precision of 2 μmol kg^–1^. For all co-sample DIC determinations, ±6 μmol kg^–1^ is used here as a maximum estimate of measurement uncertainty (SI. Table 1). Seawater TA (NOC) was determined potentiometrically with an open cell multipoint titration between pH 3.5 and 3.0^38^ using a Metrohm Ti-Touch 916 automated titrator,^42^ with a precision better than 0.1% and a combined standard measurement uncertainty of ±3 μmol kg^–1^. At BIOS, the TA was determined using the potentiometric semiclosed titration system^38^ on the VINDTA 3S, with a precision better than 0.1%. For all discrete co-sample TA determinations ±3 μmol kg^–1^ is used here as an estimate of measurement uncertainty (SI. Table 1). In addition, all discrete co-samples (*n* = 156) were analyzed for concentrations of dissolved inorganic phosphorus (hereafter phosphate) and silicic acid (hereafter silicate) at NOC following standard continuous flow analysis methods^43^ on a QuAAtro39 AutoAnalyzer (SEAL Analytical), with estimated uncertainties of 3.7 and 2.4% respectively^44^ (SI. Table 1). Finally, all discrete co-samples were analyzed for DO on the day of collection using the Winkler method^45^ following Carpenter^46^ and Langdon,^47^ on a Metrohm 794 Basic Titrino system with an estimated measurement uncertainty of 0.06%.^47^

### Sensor Measurement Validation

To validate the performance of the sensors onboard the ALR, sensor measurements were compared with the water samples collected from the ship that were analyzed using the “gold standard” laboratory techniques described above. Proximity between sensor measurements and discrete samples unavoidably varied in frequency, space, and time, making direct comparisons impossible and interpolation (a common practice when dealing with oceanographic data^48,49^) necessary. To enable effective and meaningful comparisons between sensor measurements and validation samples, the biogeochemical parameters (pH, TA, DIC, DO, S, T) measured in the discrete samples were spatially gridded using natural neighbor interpolation without extrapolation in MATLAB.^50,51^ The discrete data were interpolated rather than the sensor data to provide the most accurate representation of the water column given its trusted methodology and more consistent sampling coverage versus the ALR track. Nonetheless, the natural neighbor interpolation method was chosen because it performs well with the irregularly distributed data typically associated with oceanographic sampling.^49,52^ All parameters were regarded in *density space*, that is, they were interpolated based on their relationship to seawater density (calculated from S, T, P^53^) rather than the water depth. When parameters are compared in density space, the variability caused by vertical displacement is minimized since the ocean primarily mixes on isopycnals (density gradients) and therefore provides a clearer understanding of parameter (*x*) in question within a given water mass.^54−56^

The direct spatial comparison was achieved by extracting values from the discrete co-sample interpolant product *x*_*i*-CTD_ at the vertical (density) and lateral (location along the track) coordinates corresponding to each ALR sensor measurement *x*_ALR_. Residuals *r*(*x*) between the two measurement methods were then calculated by using eq 1.
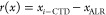
1Finally, the mixed layer depth in the DT was determined using the threshold method with criterion Δσ_θ_ = 0.125 kg m^–3^ where σ_θ_ represents potential density.^57−59^

### Carbonate System Calculations

The speciation of the carbonate system was characterized from the discrete co-sample data set obtained from the CTD casts using the CO2SYS MATLAB package^60−62^ with TA, DIC, and nutrient (phosphate and silicate) concentrations as input parameters, as well as S, T, and P from the CTD sensor on the ship’s rosette sampler. These computations yielded CTD-based pCO2 and aragonite saturation state (Ω_ar_).

The speciation of the carbonate system was also characterized by the LOC sensor data on the ALR. These data were interpolated and gridded spatially, as outlined above, to account for the differing measurement frequencies and resulting spatial and temporal mismatch between LOC sensors. The interpolated sensor TA and pH_T_ values from the generated spatial grids were used with corresponding gridded S, T, and P from the CTD onboard the ALR for carbonate system characterization using CO2SYS. All CO2SYS computations used the dissociation constants of carbonic acid (K_1_ and K_2_) from Lueker et al.,^63^ K_SO4_ from Dickson,^64^ KF from Perez and Fraga,^65^ and total Boron concentration from Lee et al.^66^ These computations produced grids of calculated carbonate system parameters (pCO_2_, DIC, and the aragonite saturation state (Ω_ar_)) from the sensor data.

The derived carbonate parameters from bottle samples (*x*_CTD_) were compared with those from the ALR sensor-derived interpolant values (*x*_*i*-ALR_) based on the corresponding vertical (density) and lateral (location along a transect) coordinates (SI. Figure 1). The resulting carbonate parameter residuals *r*_carb_(*x*) were calculated using eq 2.
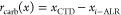
2The combined standard uncertainty *u*_C_(*y*) of *r*(*x*) was calculated as the positive square root of the combined variances using eq 3, and based on the uncertainties of sensor measurement and equivalent laboratory method (SI. Table 1).^67^
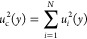
3The combined standard uncertainty of the calculated carbonate parameters was determined by error propagation using the CO2SYS function *errors.m* with the routine’s default standard errors for dissociation constant inputs^18^ and individual parameter uncertainty values from SI. Table 1. The resulting propagated error associated with each calculated carbonate parameter (for both discrete and sensor data) was used to derive the *u*_C_(*y*) of *r*_carb_(*x*) using eq 3 and values from SI. Table 1.

## Results and Discussion

### Autonomous Data Collection

During March 20–29, 2022, the ALR successfully completed over 10 dive missions that formed the Shelf Transect (ST) and the Deep Transect (DT). The pH, TA, and DIC Lab-On-Chip sensors onboard the ALR made 947, 423, and 251 in situ measurements, respectively. Data taken from the ALR Lab-On-Chip sensors for pH_T_ (pH_T-ALR_) and TA (TA_ALR_) are used in this study. During the same period, 156 discrete water samples were taken from the water column and analyzed for TA (TA_CTD_) and DIC (DIC_CTD_), 47 of which were also analyzed directly for pH_T_ (pH_T-CTD_). The CTD-DO sensors on the ALR and ship-based CTD rosette sampler recorded continuous (1 Hz) measurements for salinity (S_ALR_ and S_CTD_), temperature (T_ALR_ and T_CTD_), pressure (P_ALR_ and P_CTD_), and dissolved oxygen (DO_ALR_ and DO_CTD_). Values of pH_T_, TA, DIC, S, and T collected from sensors and bottle samples fell within the expected ranges for the region and generally agreed with each other (Table 2).

**Table 2:** Summary of the Observed and Historical Parameter Values^a^

parameter	ALR transects	CTD casts	regional data^1^	regional data^2^
pH_T_	7.97–8.09	7.94–8.06	8.1–8.2	n/a
TA (μmol kg^–1^)	2302–2370	2314–2357	2310–2360	2326–2345*
DIC (μmol kg^–1^)	2107–2182	2117–2217	2050–2150	2074–2135*
salinity (PSU)	35.34–35.61	35.34–35.60	34.4–n/a	35.04–35.47
temperature °C	10.56–12.29	10.32–12.36	7.5–12.5	8.8–13.9

a ^1^Kitidis et al.^68^ Western English Channel (Stations E1 & L4 Feb–April). ^2^Marrec et al.^69^ Western English Channel (Stations E1 & L4 Spring). *Indicating surface water measurements only.

The prototype DIC sensor operated throughout the majority of the deployment and produced measurements within expected ranges (Table 2). However, DIC observations did not follow expected trends (i.e., increase with depth due to carbon mineralization) and rather showed random variability over the deployments. Investigation postdeployment pointed toward failure of the gas exchange unit and calibration error; therefore, sensor DIC data were flagged as unreliable and will not be discussed further. Since the deployments described here, a new version of the DIC LOC sensor has been developed and is undergoing field trials.

### Comparison between Ship-Based and Autonomous Observations

#### General Hydrography and Biogeochemistry

One of the primary objectives of this study was to evaluate whether an autonomous observing system such as the one described here could provide information comparable to that of traditional shipside collection along oceanographic transects. The observational plan was therefore designed to enable meaningful comparisons between the ALR and ship observations. Validation bottle sample collection was planned along the programmed ALR path and (where possible) at a time when the ALR was in proximity but at a safe distance to avoid collision with the CTD rosette. The time between ALR sensor measurements and bottle sample collection in the same proximity ranged between 1 min and 15 h (μ = 6 h) for the ST and between 5 min and 85 h (μ = 40 h) for the DT.

Co-location of sensor measurements and validation samples is more critical in shallow waters (within the mixed photic zone) due to light-driven diel biogeochemical variability, irregularity in phytoplankton abundance, and strong mixing from tidal currents, which also affect biogeochemical variables. Most observations collected along the ST by the ALR were either within the vertically homogeneous (mixed) waters above the continental shelf or within the ≈320 m surface mixed layer off the shelf. Both ALR and ship-based observations show similar trends along the ST. There is a lateral gradient of increasing salinity (35.35–35.55 PSU) and temperature (11–12.5 °C) as the transect moves away from the continental shelf (SI. Figure 2). This trend is consistent with freshwater influence from the coast and the existence of a warmer mixed layer offshore. Dissolved oxygen decreased with depth (260–220 μmol kg^–1^), with peaks (275 μmol kg^–1^) near the surface (60–100 km along the track) where elevated fluorescence concentrations (1.1 μg L^–1^) were detected consistent with primary productivity (SI. Figure 2).

There was closer agreement between ship and ALR observations along the DT where vertical stratification was present. The mixed layer depth-averaged 345 m calculated from ship CTD observations, which is similar to that calculated using the ALR observations (338 m). Seawater salinity ranged from 35.45 to 35.6 PSU, the temperature ranged from 10.5 to 12.4 °C, and the DO ranged from 180 to 245 μmol kg^–1^ (SI. Figure 3). It is important to note that because of the sampling spatiotemporal differences between the ALR and CTD, their water mass properties are not identical. While it is evident that each of the ALR and CTD salinity, temperature, dissolved oxygen, and density observations show the same general trends and ranges in each of the sampling transects, there are still subtle differences that are reflected in and propagated through their respective carbonate measurements and comparison residuals.

#### pH

**Figure 5:**
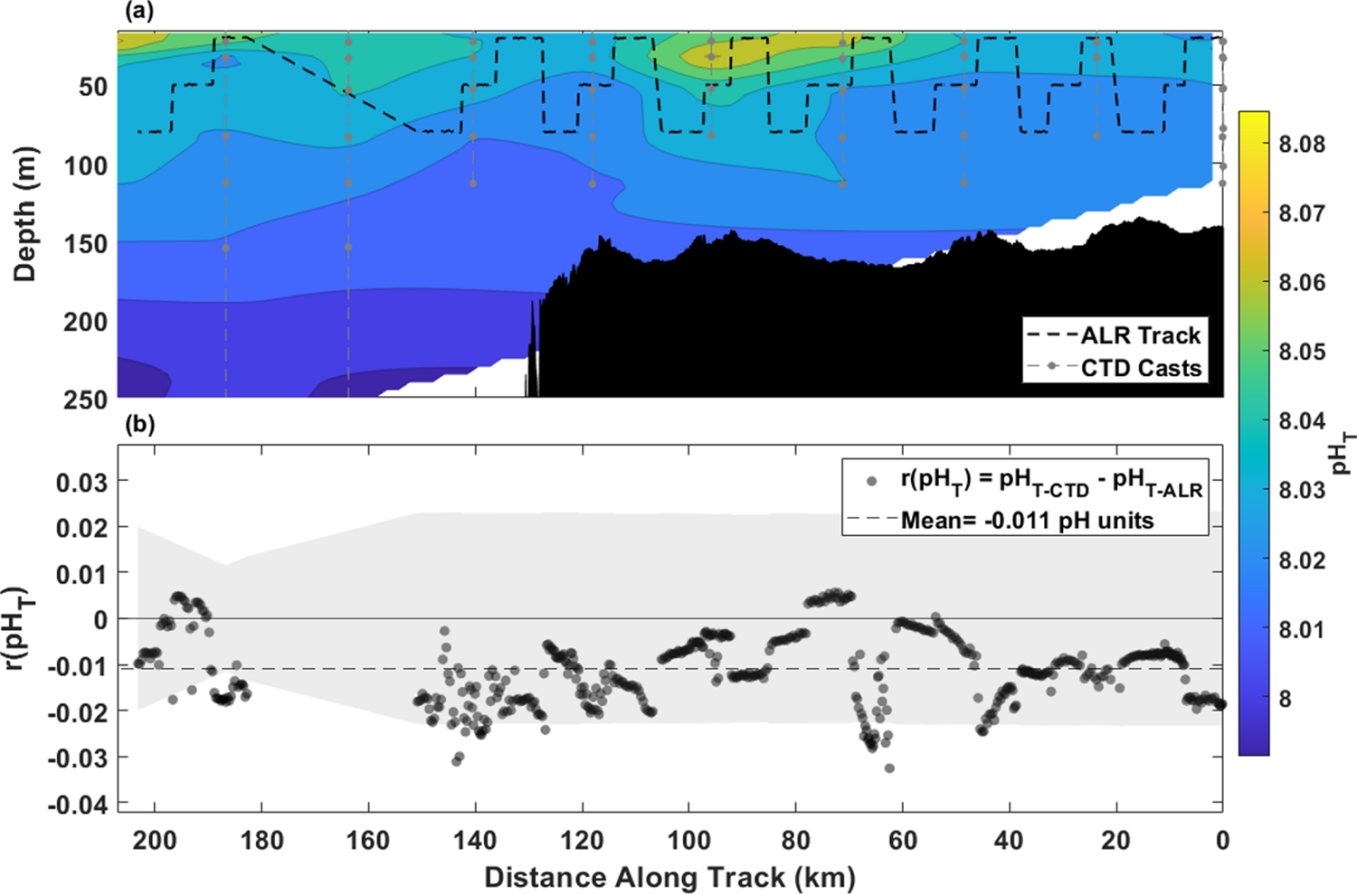
Shelf Transect pH_T_ and intercomparison. The shared *x*-axis is plotted from right to left to better represent the geographic location and direction of travel (away from the continental shelf). (a) Contour map from interpolated pH_T-CTD_ with contours denoted by color with respect to depth (m) on the *y*-axis and distance (km) on the *x*-axis. (b) Residuals of pH_T-CTD_ – pH_T-ALR_ plotted where pH_T-CTD_ is an interpolated value at the density and distance where pH_T-ALR_ measured. Running combined standard uncertainty (mean = ±0.022) is shaded in gray.

The pH_T-ALR_ data show good agreement with interpolated pH_T-CTD_ across both transects (Figures 5 and 7). During the ST, pH_T_ values ranged 0.094 pH units from 7.991 to 8.085 throughout the water column. Generally, pH decreased with depth, with the highest values recorded in regions of high fluorescence and oxygen concentrations (Figure 5a and SI. Figure 2). A strong positive correlation (*n* = 950, *R*^2^ = 0.87, SI. Figure 4) between pH_T_ and DO throughout the deployment implies a primarily biological control (photosynthesis–respiration) on pH variability within the surface mixed layer.

The mean residual between interpolated pH_T_ from bottle measurements and pH_T-ALR_ (*r*(pH_T_)) in density space along the ST was −0.011 (σ = 0.008, *n* = 560). *r*(pH_T_) varied from −0.033 to 0.006 with 94% of *r*(pH_T_) within the mean combined standard uncertainty (±0.022) of the sensor and lab-based pH analysis (Figure 5b). The negative bias in *r*(pH_T_) reflects the consistently higher pH_T-ALR_ values than pH_T-CTD_. The source of this systematic bias is likely the ALR pH sensor measurements, as it would be highly improbable for calculated pH_T-CTD_ and directly measured pH_T-CTD_ analysis to carry the same bias. Further investigation into the pH sensor’s raw data revealed no signs to suggest that its performance was compromised. The pH sensor’s thermistors and optics were functioning correctly, indicating that the observed bias was not due to equipment limitations, but rather likely a deployment-related reason. This points to another notable challenge when comparing ocean observation methodologies and necessitates further research.

**Figure 6:**
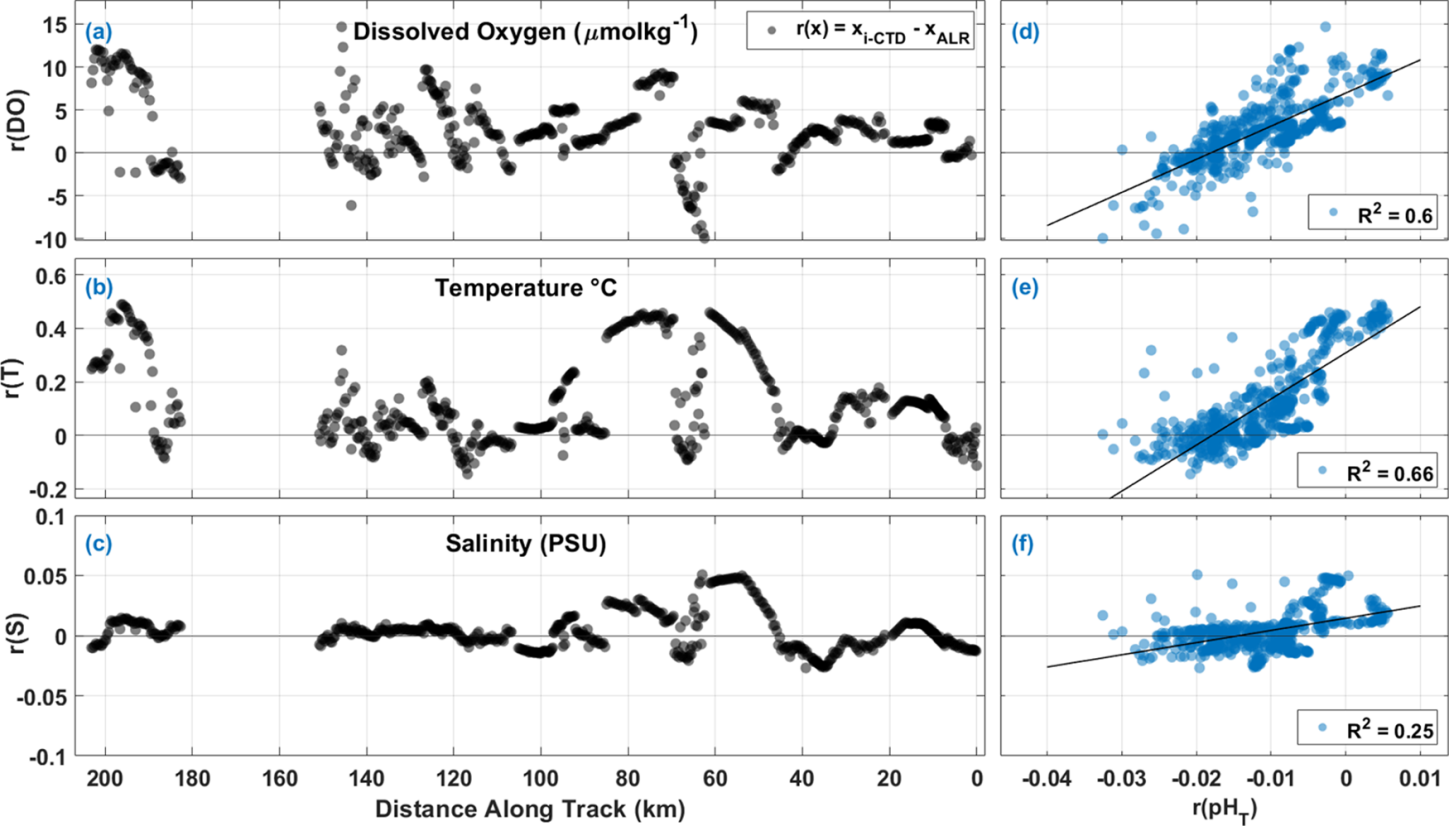
Comparison of residuals from measurements of dissolved oxygen, temperature, and salinity in the ST with respect to distance (km) along transect (a–c) and *r*(pH_T_) (a–f). (a) Residuals of dissolved oxygen *r*(DO) in μmol kg^–1^ along the distance. Gray circles represent residuals calculated as *r*(*x*) = *x*_*i*-CTD_ – *x*_ALR_. (b) Residuals of temperature *r*(T) in °C and (c) residuals of salinity *r*(S) in PSU along the distance, with the same legend as in (a). (d) *r*(DO) as a function of pH_T_ residuals *r*(pH_T_). (e) *r*(T) as a function of *r*(pH_T_). (f) *r*(S) as a function of *r*(pH_T_).

To help interpret the *r*(pH_T_) in the ST, residuals from other observed parameters such as DO, S, and T were also evaluated in the density space along the transect (Figure 6). For residuals of DO (*r*(DO)), S (*r*(S)), and T (*r*(T)) evaluated along the ST, the larger deviations from *r*(*x*) = 0 (where *x*_*i*-CTD_ = *x*_ALR_), coincided in space with larger *r*(pH_T_) as illustrated clearly between ≈40 and 80 km along the ST track (Figure 6a–c). In fact, *r*(pH_T_) correlated positively with *r*(DO), *r*(S), and *r*(T) [*r*(DO)/*r*(pH_T_) (*R*^2^ = 0.60, *p* < 0.001), *r*(S)/*r*(pH_T_) (*R*^2^ = 0.25, *p* < 0.001, and *r*(T)/*r*(pH_T_) (*R*^2^ = 0.66, *p* < 0.001)] in density space, along the entire transect as shown in Figure 6d–f.

Similar comparisons between *r*(pH_T_) and depth, as well as *r*(pH_T_) and hours between sample collection versus sensor measurement (*r*(Time)), show a weak yet significant correlation (*p* < 0.001, SI. Figure 5). The highest *r*(pH_T_) values correspond to the highest *r*(time) (5–15 h), and especially where (at ≈60–80 km along the ST track) fluorescence was highest (areas of high primary productivity) (SI. Figure 2). This implies that in highly productive waters, such as the shelf region of the Celtic Sea on the cusp of spring, spatiotemporal variability in biogeochemistry makes measurement comparisons between platforms and sensor measurement validation challenging. Therefore, sensor measurement validation should be avoided in shallow productive waters or special care must be taken to minimize the spatiotemporal mismatch between sensor measurements and validation samples.

**Figure 7:**
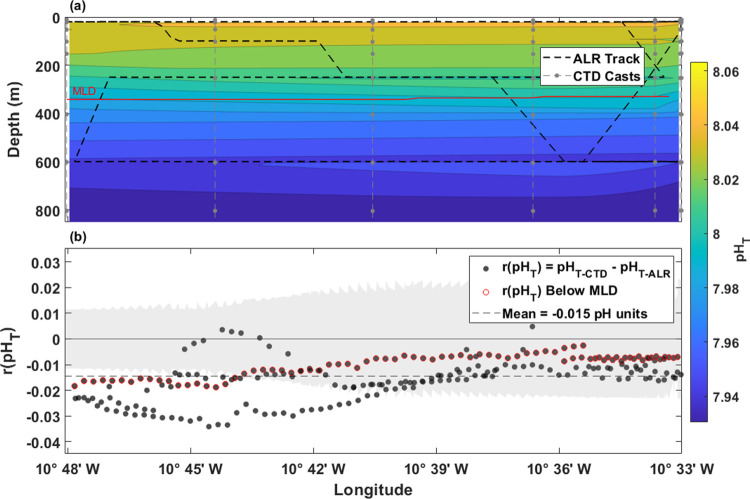
Deep Transect pH_T_ data and intercomparison. Shared *x*-axis represents longitudinal position on transect. (a) Contour map created from interpolated pH_T-CTD_, and contours denoted by color with respect to depth (m) on the *y*-axis and longitude on the *x*-axis. (b) Residuals of pH_T-CTD_ – pH_T-ALR_ plotted where pH_T-CTD_ is an interpolated value at the density and longitude where pH_T-ALR_ was measured. The r(pH_T_) values calculated from observations below the average mixed layer depth (MLD) of 345 m are outlined in red. Running combined standard uncertainty (mean = ±0.018) is shaded in gray.

Along the DT, observed pH_T_ values ranged between 7.931 in deep waters (1000 m) and 8.063 close to the surface (Figure 7a). The observed pH_T_ decreased uniformly with depth, reflecting the shift from net photosynthesis to net respiration with diminishing light availability.

The mean *r*(pH_T_) in the DT was −0.015 (σ = 0.008, *n* = 234) with a similar negative bias as seen in the ST. Values of *r*(pH*_T_*) varied from −0.034 to 0.005 with 77% of *r*(pH_T_) within the mean combined standard uncertainty (±0.018) of the sensor and lab-based pH analysis (Figure 7b).

**Figure 8:**
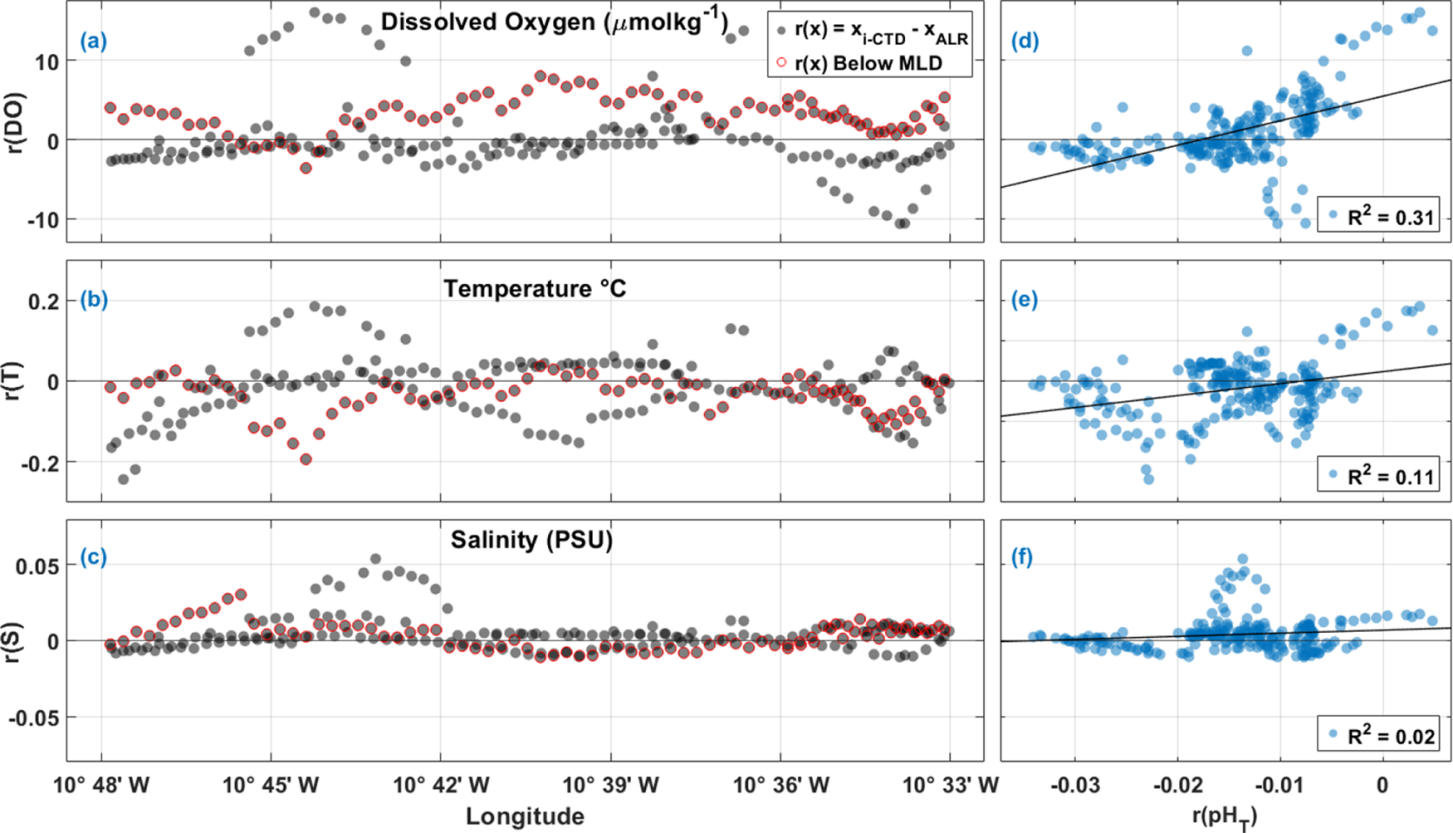
Comparison of residuals from measurements of dissolved oxygen, temperature, and salinity in the DT with respect to longitude (a–c) and *r*pH_T_ (d–f) in the DT. (a) Residuals of dissolved oxygen *r*(DO) in μmol kg^–1^ along longitude. Gray circles represent residuals calculated as *r*(*x*) = *x*_*i*-CTD_ – *x*_ALR_, of those residuals, outlined in red are below the average mixed layer depth (MLD) of 345 m. (b) Residuals of temperature *r*(T) in °C and (c) Residuals of salinity *r*(S) in PSU as along longitude, with the same legend as in (a). (d) *r*(DO) as a function of *r*(pH_T_). (e) *r*(T) as a function of *r*(pH_T_). (f) *r*(S) as a function of *r*(pH_T_).

Along longitude in the DT, particularly between 10°42′ W and 10°45′ W, there are notable differences between the ALR and CTD DO, S, and T that mimic the larger spread of pH_T_ residuals at the same location (Figure 8a–c). The *r*(pH_T_) values correlated positively with *r*(DO) (*R*^2^ = 0.31, *p* < 0.001) and *r*(T) (*R*^2^ = 0.11, *p* < 0.001), although not with *r*(S) (Figure 8). There was no significant relationship between *r*(Time) and *r*(pH_T_) (SI Figure 6). Interestingly, depth correlated positively with *r*(pH_T_) in the DT (*R*^2^ = 0.30), showing more pH_T_ variability closer to the surface rather than at depth (SI. Figure 6d). Unlike pressure effects commonly seen with oceanographic instrumentation use, the trend seen here comes from a mismatch near the surface and within the MLD. As seen in Figures 7b and 8a–c, data points that are below the MLD (outlined in red) showed slightly more stability than those above the MLD. This reflects the challenge in comparing biogeochemical observations made in heterogeneous waters such as productive regions or across physical boundaries such as nutriclines and fronts.

Similar to the ST pH intercomparison, there is evidence here that the ALR was operating in biogeochemically different water than the CTD casts at times, which then led to pH disagreement. However, the negative bias of the pH residuals is likely not a coincidence and may point to other operational and systematic insights, as described earlier about the ST.

In both transects, the pH LOC sensor measurements showed overall good agreement with the pH_T_ of the CTD discrete samples with maximum r(pH_T_) in the order of 0.035 which is twice as large as previously reported for this device (−0.013 by Yin et al.^27^; 0.015 by Nehir et al.^70^) in shallow coastal water deployments. The largest discrepancy between pH_T-ALR_ and pH_T-CTD_ reported here likely reflects the temporal and spatial mismatch between the sensor measurements and the CTD sample collection. The pH LOC sensor has previously been integrated on a Seaglider and deployed in the North Sea for a duration of 10 days. During the deployment, four samples were taken using the ship’s CTD alongside the glider and analyzed for TA and DIC. The calculated pH_T_ was within 0.005 from the sensors' pH_T_ measurements. However, examples of pH observations from AUVs are very limited and are still at the “experimental” stage.^71,72^ This is mainly because, unlike the pH LOC sensor, no other technology is readily integrable on small platforms (such as gliders), with most requiring bespoke electronics and housings. More recently the “Deep-Sea DuraFET” pH sensor (based on Honeywell’s Durafet ISFET technology^73^) has been integrated and demonstrated on a Spray glider with promising results.^74^ The mean difference between sensor pH_T_ measurements and pH_T_ measured in CTD samples using spectrophotometry was on the order of 0.006 ± 0.021 (*n* = 155). The only commercially available stand-alone Deep-Sea DuraFET sensor (SeaFET/SeapHOx, Seabird Scientific) however, is designed mainly for moored applications. Both spectrophotometric and ISFET-based technologies show promise for ocean carbon observations,^27,75^ yet more work is needed to improve analytical performance in order to meet GOOS requirements and enable easy integration by end-users on autonomous platforms.

#### Total Alkalinity

**Figure 9:**
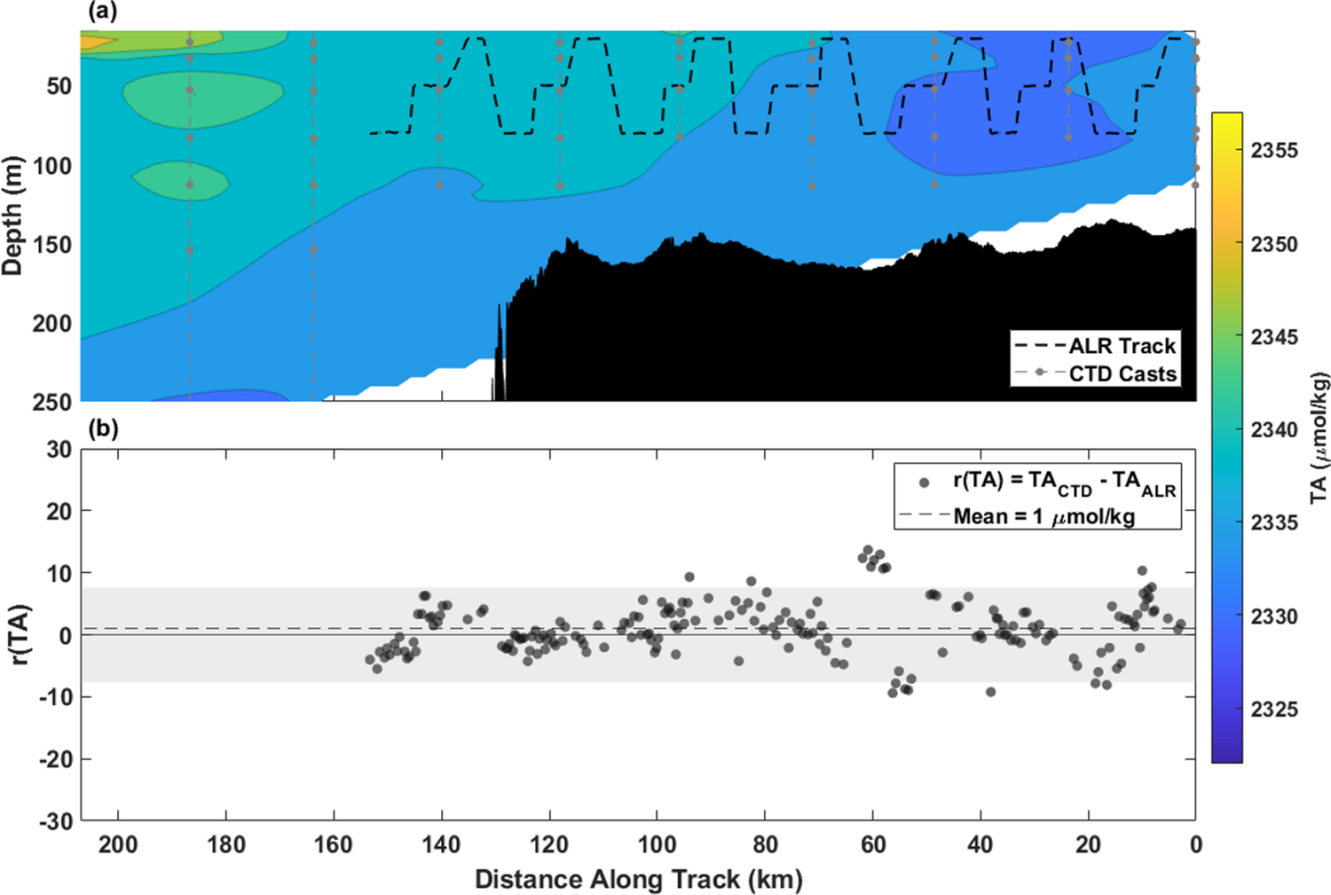
Shelf Transect TA and intercomparison. The shared *x*-axis is plotted from right to left to better represent geographic location and direction of travel (away from the continental shelf). (a) Contour map created from interpolated TA_CTD_ and contours denoted by color with respect to depth (m) on the *y*-axis and distance (km) on the *x*-axis. (b) Residuals of TA_CTD_ – TA_ALR_ plotted where TA_CTD_ is an interpolated value at the density and distance where TA_ALR_ measured. Running combined standard uncertainty (mean = ±8 μmol kg^–1^) is shaded in gray.

Total alkalinity measured in situ by the TA sensor onboard the ALR (TA_ALR_) agreed very well in density space with the TA measured in the bottle samples collected from the ship’s CTD (TA_CTD_) along both the ST and DT. Along the ST, the observed TA values ranged between 2314 and 2357 μmol kg^–1^ throughout the 250 m water column (Figure 9a). There was a horizontal gradient of increasing TA away from the UK coast as with salinity (SI. Figure 2b), consistent with the influence of low-TA freshwater inputs from land.^76^ The mean *r*(TA) was 1 μmol kg^–1^ (σ = 4, *n* = 191), ranging between −9 and 14 μmol kg^–1^ with no observed bias. Of the TA residuals along ST, 91% fall within the mean combined uncertainty (±8 μmol kg^–1^) of the sensor and lab-based TA analysis (Figure 9b).

**Figure 10:**
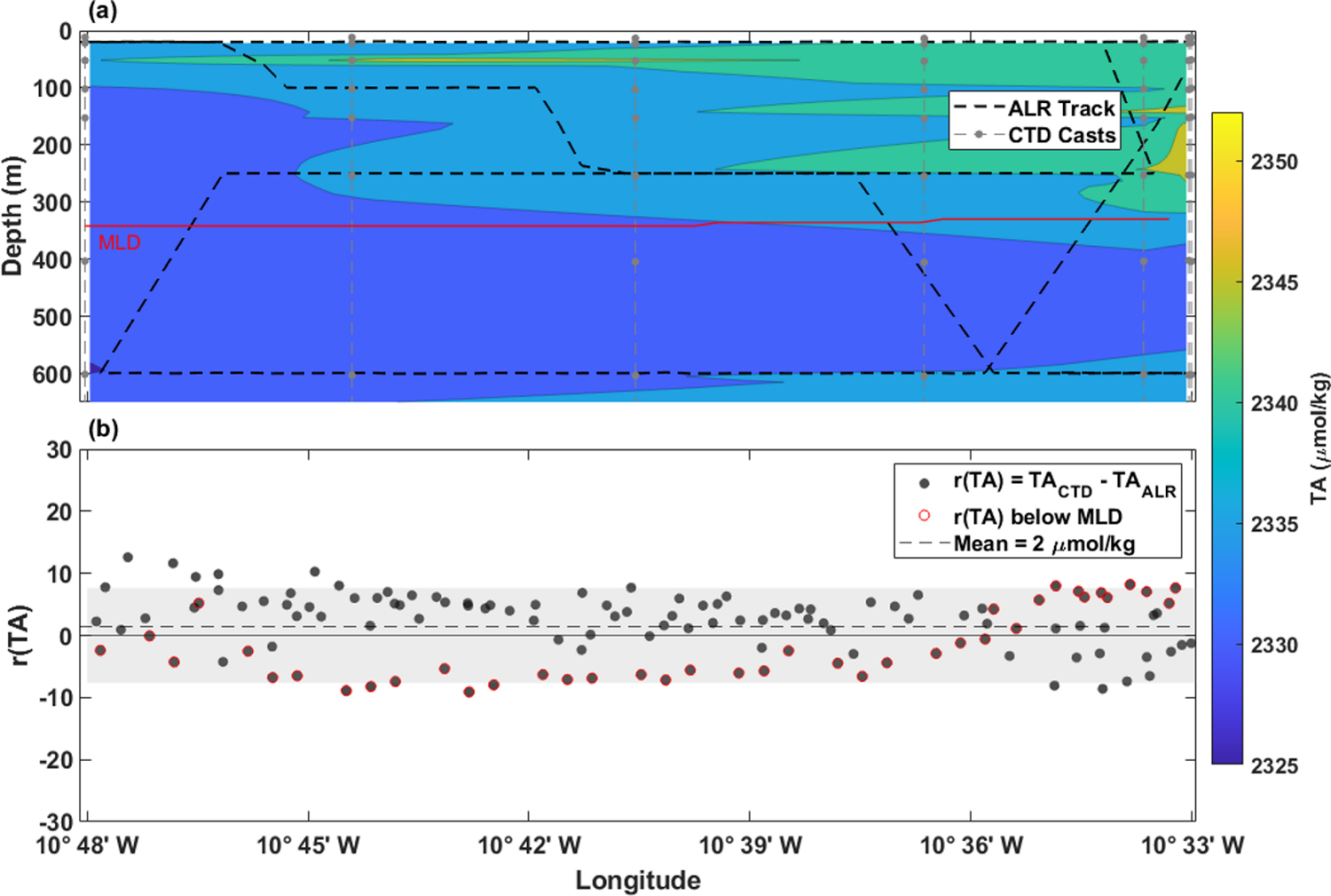
Deep Transect TA data and intercomparison. (a) Contour map created from interpolated TA_CTD_ and contours denoted by color with respect to depth (*m*) on the *y*-axis and longitude on the *x*-axis. (b) Residuals of TA_CTD_ – TA_ALR_ plotted where TA_CTD_ is an interpolated value at the density and longitude where TA_ALR_ measured. The *r*(TA) values calculated from observations below the mean mixed layer depth (MLD) of 345 m are outlined in red. Running combined standard uncertainty (mean = ±8 μmol kg^–1^) is shaded in gray.

Along the DT, the observed TA ranged between 2325 and 2352 μmol kg^–1^ throughout the 600 m of the sampled water column (Figure 10a). The mean *r*(TA) was 2 μmol kg^–1^ (σ = 5, *n* = 129), and ranged between −9 and 13 μmol kg^–1^ with no observed bias; 87% of *r*(TA) falls within the bounds of the mean combined uncertainty (±8 μmol kg^–1^) of the sensor and lab-based TA analysis (Figure 10b). The good agreement between TA*_ALR_* and TA*_CTD_* also reflects the spatial and temporal homogeneity with respect to TA which exhibits a largely conservative distribution in the open ocean and is not significantly affected by biological processes.

According to current research, this is the first demonstration of a TA sensor in an autonomous vehicle. Other autonomous TA sensors based on similar spectrophotometric technology are reported in the literature, however, they are currently limited to shallow-moored applications.^77,78^ The NOC LOC platform, on which the TA LOC sensor is based (as are the pH and DIC LOC), is specifically designed for integration versatility on stationary and small moving platforms. Although TA can be estimated with a certain confidence in large parts of the ocean,^20^ direct measurements are necessary to accurately constrain TA and the seawater–carbonate system in regions with high calcification or riverine inputs.^79,80^ Additionally, TA sensors will be a key tool in quantifying TA generation and dynamics at Ocean Alkalinity Enhancement (OAE) application sites.

### Constraining the Marine Carbonate System Autonomously

To our knowledge, this work presents the first in situ characterization of the marine carbonate system from an autonomous vehicle based on direct sensor measurements of pH_T_ and TA. The challenge so far in achieving this has been the lack of in situ sensors (mainly for TA and DIC) capable of autonomous observations onboard moving platforms such as AUVs, ASVs, and floats. For this reason, autonomous characterization of the carbonate system (e.g., BGC Argo and SOCCOM programs) currently relies on measured pH and modeled TA, which carries uncertainties. Although in principle the carbonate system can be measured and constrained using commercially available pH and pCO_2_ sensors (such as the PMEL mooring-based observing network^81^), this approach uses the least desirable combination of input variables due to large errors propagated in the calculated carbonate system parameters.^18,19^ Additionally, pCO_2_ sensors are much less practical on moving platforms due to technical limitations including slow response times, integrability, and high power requirements.^82^

In the following sections, we present carbonate system parameters calculated from pH_T-ALR_ and TA_ALR_, including dissolved inorganic carbon (DIC_ALR_) in μmol kg^–1^, partial pressure of carbon dioxide (pCO_2-ALR_) in μatm, and aragonite saturation state (Ω_ar-ALR_) which is unitless. We then compare these parameters against those calculated from measured TA_CTD_ and DIC_CTD_ (pCO_2-CTD_ and Ω_CTD_) in order to evaluate the capability of the ALR-sensor system to constrain the seawater–carbonate system.

#### Shelf Transect

**Figure 11:**
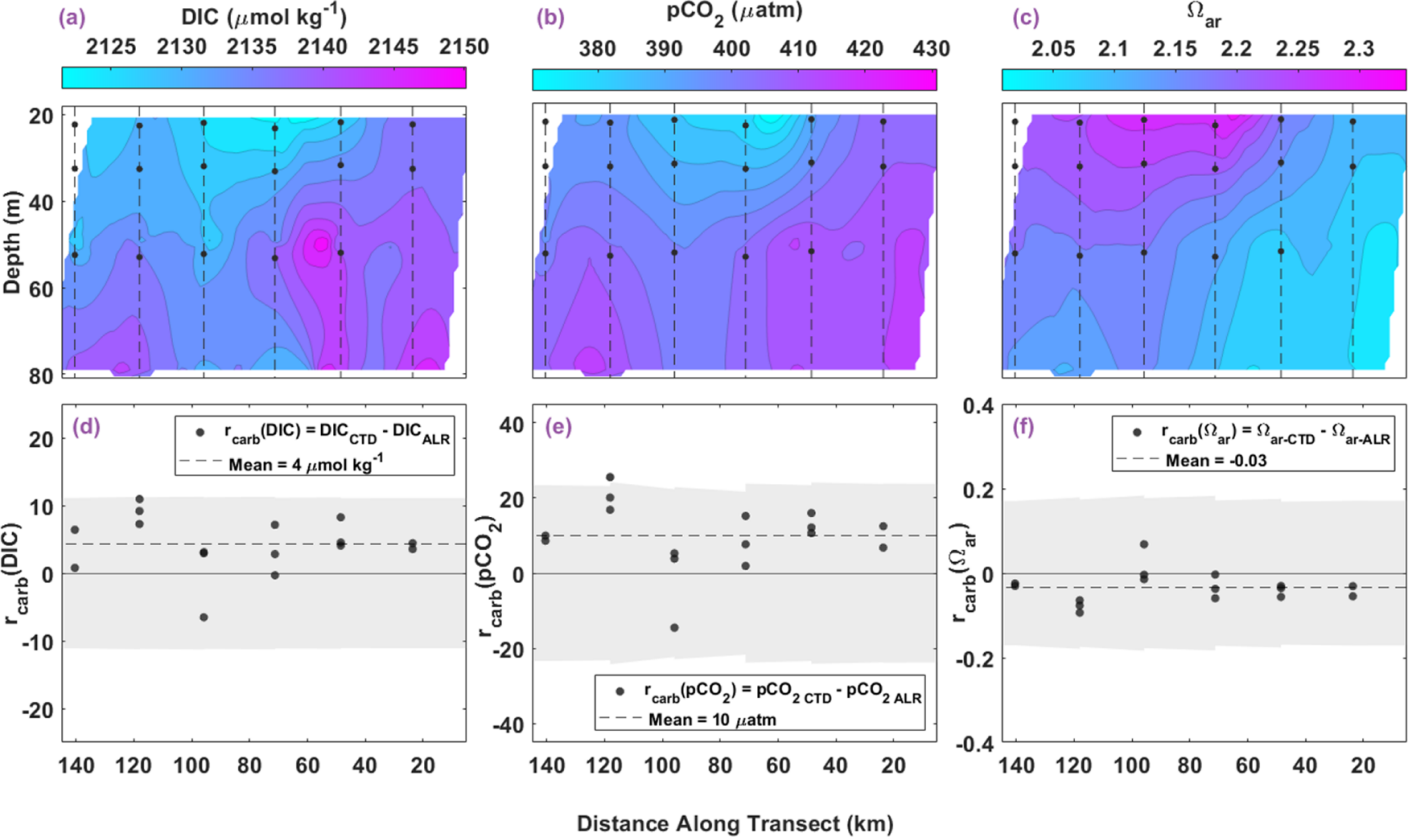
Vertical contoured distribution of calculated carbonate system parameters from ALR sensor data in the ST (a–c), paired with comparison measured and calculated bottle sample carbonate parameters (d–f). (a) Contour of DIC_ALR_ (μmol/kg) in the ST water column along the transect distance. Black circles connected by dashed lines represent bottle sample locations from CTD casts. (b) Contour of pCO_2-ALR_ (μatm) and (c) contour of Ω_ar-ALR_ with the same format as in (a). Residuals show variability between CTD and ALR measurements along ST, where *x*_*i*-ALR_ is an interpolated value at the density and distance where *x*_CTD_ was collected, to produce residual *r*_carb_(*x*). (d) Residuals of DIC (*r*(DIC), μmol/kg) along the transect distance (km). Black circles represent residuals calculated as *r*(DIC) = DIC_CTD_ – DIC_ALR_. The dashed line indicates the mean residual (4 μmol/kg). (e) Residuals of pCO_2_ (*r*(pCO_2_), μatm) along the track distance with mean residual of 10 μatm, and (f) residuals of Ω_ar_ (*r*(Ω_ar_)) as a function of longitude with mean residual of −0.03, both with the same legend as in (d) for respective parameters. Gray shaded regions in plots (d–f) represent running combined standard uncertainty, including error propagation, with means of (d) ±11 μmol, (e) ±23 μatm, and (f) ±0.18.

The sample area for constraining the carbonate system on the shelf is a subset of the ST used in the above analysis, dictated by where both pH_T-ALR_ and TA_ALR_ measurements were available. It spans 0–140 km along the transect and is from 20–80 m in the water column (SI. Figure 5). Calculated DIC_ALR_ ranged from 2121 to 2150 μmol kg^–1^ and pCO_2-ALR_ ranged from 366 to 431 μatm with higher values for both at depth. Calculated Ω_ar-ALR_ ranged from 2.4 near the surface to 2.0 at depth (Figure 11a–c). The variability observed in measured pH_T-ALR_ close to the surface (likely caused by primary productivity) propagated into the calculated DIC_ALR_, pCO_2-ALR_, and Ω_ar-ALR_.

When compared in density space, the directly measured DIC_CTD_ and the calculated DIC_ALR_ show very good agreement with a mean *r*(DIC) of 4 μmol kg^–1^ (σ = 4, *n* = 16), and 100% of the residuals within the mean combined uncertainty of ±11 μmol kg^–1^ (Figure 11a,d). Similarly, there is also good agreement between the calculated pCO_2-CTD_ and pCO_2-ALR_. The mean *r*(pCO_2_) is 10 μatm (σ = 9, *n* = 16), where 94% of the residuals lie within the mean combined analytical uncertainty of ±23 μatm (Figure 11b,e).

The mean *r*(Ω_ar_) is −0.03 (σ = 0.04, *n* = 16) with 100% of the residuals within the mean combined uncertainty of ±0.18 (Figure 11c,f). The positive bias in the ST DIC and pCO_2_ residuals and the negative bias in Ω_ar_ residuals reflect the bias in pH_T-ALR_ propagated through the carbonate system calculations, as mentioned earlier.

#### Deep Transect

The interpolated grid for carbonate calculations of the DT uses the entire same sample area as that in the previous section. Along the DT, measured and calculated DIC ranged from 2123 to 2190 μmol kg^–1^, calculated pCO_2_ ranged from 388 to 551 μatm, and calculated Ω_ar_ ranged from 1.5 to 2.3. Similar to the shelf region, Ω_ar_ decreases with depth as a consequence of the higher DIC and pCO_2_ concentrations in deeper waters.

**Figure 12:**
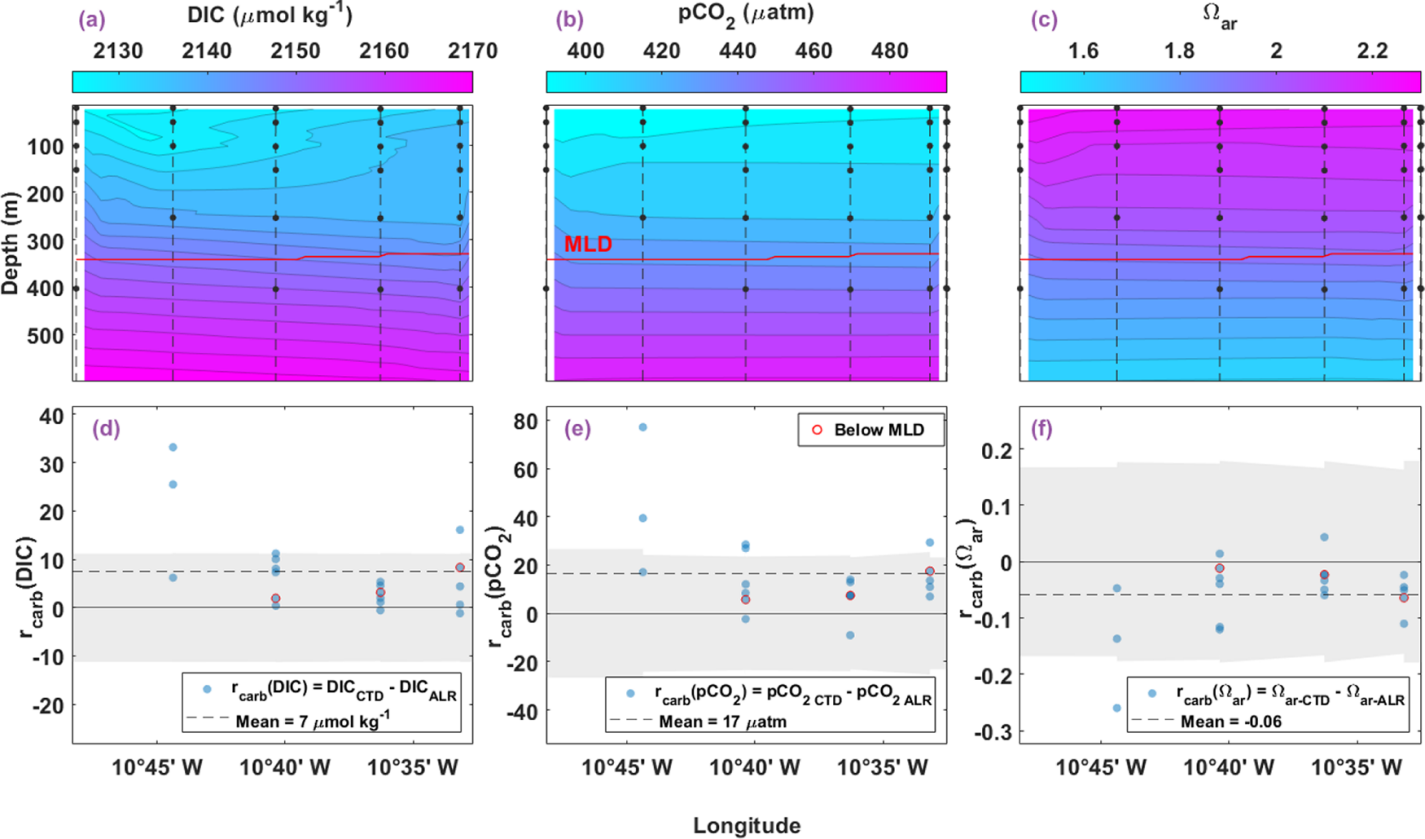
Vertical contoured distribution of calculated carbonate system parameters from ALR sensor data in the DT (a–c), paired with comparison of measured and calculated bottle sample carbonate parameters (d–f). (a) Contour of DIC_ALR_ (μmol/kg) in the DT water column along the longitudinal transect. Black circles connected by dashed vertical lines represent bottle sample locations from CTD casts. Red horizontal line indicates the average MLD at 345 m. (b) Contour of pCO_2-ALR_ (μatm) and (c) contour of Ω_ar-ALR_ with the same format as in (a). Residuals show variability between CTD and ALR measurements along DT, where *x*_*i*-ALR_ is an interpolated value at the density and longitude where *x*_CTD_ was collected, to produce residual *r*_carb_(*x*). (d) Residuals of DIC (*r*_carb_(DIC), μmol/kg) along the longitude. Black circles represent residuals calculated as *r*_carb_(DIC) = DIC_CTD_ – DIC_ALR_. Circles outlined in red represent residuals below the MLD. The dashed line indicates the mean residual (7 μmol/kg). (e) Residuals of pCO_2_ (*r*_carb_(pCO_2_), μatm) along longitude with mean residual of 17 μatm, and (f) residuals of Ω_ar_ (*r*_carb_(Ω_ar_)) along longitude with a mean residual of −0.06, both with the same legend as in (d) for respective parameters. Gray shaded regions in bottom plots represent running combined standard uncertainty, including error propagation, with means of (d) ±11 μmol, (e) ±24 μatm, and (f) ±0.17.

For calculated carbonate system evaluation in the DT, the median (*x̃*) residual is also reported because the mean residual is skewed from comparisons near 10°45′ W (Figure 12d–f). Compared in density space, the measured DIC_CTD_ and the calculated DIC_ALR_ in the DT result in a mean residual of 7 μmol kg^–1^ (σ = 9 *n* = 20, *x̃* = 5). 85% of *r*_carb_(DIC) lie within the mean combined uncertainty of ±11 μmol kg^–1^ (Figure 12d). Between calculated pCO_2_ from both the ALR and CTDs, mean *r*_carb_(pCO_2_) is 17 μatm (σ = 18, n = 20, *x̃* = 13) where 75% of the residuals lie within the uncertainty region of ±24 μatm (Figure 12e). Finally, the mean *r*_carb_(Ω_ar_) is −0.06 (σ = 0.06, *n* = 20, *x̃* = −0.05). Of the Ω_ar_ residuals in the DT, 95% are within the combined standard uncertainty region spanning on average ±0.17 (Figure 12f). As mentioned previously, there are large disparities between the ALR and CTD carbonate parameters along the DT near 10°45′ W that were likely amplified as they propagated through the carbonate calculations. Divergences in *r*(S), *r*(T), *r*(DO), and *r*(pH_T_) are in the same location (10°45′ W) of the DT (Figures 7b and 8a–c). This shows that each input parameter—and their differences—contribute to calculated carbonate variable final values. For all residuals of calculated carbonate variable comparisons within the DT, those that lie below the MLD (outlined in red in Figure 12d–f) are the smallest, reflecting the higher biogeochemical homogeneity at depths where biological processes are less dominant.

Overall, carbonate system parameters calculated using the ALR-sensor pH and TA are comparable to those calculated from the CTD sample measurements with most residuals within the expected combined analytical uncertainty. This does not only add confidence to the quality of the sensor measurements but also to the data treatment used (i.e., spatial interpolation) to enable carbonate system characterization. It is important to note that the residuals calculated from comparisons between ALR and CTD observations reflect not only the analytical uncertainty of the sensor and laboratory measurements but also the spatiotemporal mismatch between the two. Measurement uncertainties of pH_T_ and TA from both sensors and discrete bottled samples met GOA-ON’s weather quality objective (0.02 for pH_T_, 10 μmol kg^–1^ for TA) but not the climate quality objective (0.003, 2 μmol kg^–1^).^83^ Similarly, the uncertainty in DIC measurements—both directly from bottled samples and as propagated error from CO2SYS—met the weather quality objective (10 μmol kg^–1^) but fell short of the climate quality standard (2 μmol kg^–1^).^83^ The presented work suggests that the autonomous technology described here can provide viable carbonate system information along transects, as has been traditionally done so far from ships. However, further work is necessary to improve the analytical performance of the autonomous sensors, in order to match the measurement quality that can be achieved through laboratory analysis and satisfy EOV quality objective requirements such as GOA-ON’s.

Considerations must also be paid to sensor operation and specifically measurement synchronization. The carbonate sensors on the ALR were configured to make measurements at their maximum measurement frequency, as illustrated in Table 1. A 2.5 min difference between the pH LOC and TA LOC measurements may seem small but it translates into a spatial mismatch in the order of around 100 m. Traditionally, the carbonate system is characterized by measurements (usually TA and DIC) of the same sample. In the case of the ALR observations, the mismatch was addressed by gridding the TA_ALR_ and pH_T-ALR_ data and using the resulting compatible arrays for carbonate system calculations. Although this is a valid approach in accounting for this issue,^48,49,52^ special care must be taken when applying this treatment in waters with high spatial biogeochemical variability such as productive surface waters, fronts, and sharp vertical gradients. Our data demonstrates that comparisons between ALR and CTD measurements can be challenging in productive surface waters when there is a sample measurement mismatch in space and time. Inevitably, carbonate system parameters calculated from interpolated values will carry uncertainty that is difficult to quantify. To avoid this issue, it is, therefore, recommended that sensors are configured so that measurements coincide as much as possible, especially when monitoring in biogeochemically heterogeneous waters.

### Future Perspectives

To this day, ship-based hydrography remains the only method for obtaining high-quality carbonate system data over the full ocean column. Global hydrographic surveys have been carried out across defined transects (e.g., GO-SHIP^84^) approximately every decade since the 1970s, and they are the primary source of information on the status and changes to the ocean carbon system. The advancement of AUVs equipped with carbonate sensors presents a new breakthrough in dynamic observing, monitoring, and characterizing the marine carbonate system at high spatiotemporal resolutions. This high-resolution data is necessary to understand fine-scale processes and localized high-frequency changes in pH and CO_2_ fluxes. The work presented here demonstrates that autonomous ocean-observing technology is reaching a readiness level where it can generate carbon observations currently only possible using ships. As it stands, AUV endurance cannot cover the longest of the GO-SHIP repeat transects (on the order of 10,000 km), while sensor analytical performance also needs to be improved. The LOC sensors are designed to be integrated into other platforms. The key consideration for integration is the availability of supplementary data (S, T, and P) required for parameter determination by the sensor. Following this, the primary limiting factor for LOC sensors in these types of autonomous deployments is power demand, whereas in systems with continuous power (e.g., underway ship systems or cabled arrays), reagent supply and occasional mechanical or electronic failures become more critical limitations. Optimal sensor performance requires balancing sampling frequency with deployment duration, site conditions, power availability, and spatial constraints to effectively manage reagent storage, waste disposal, mechanical wear, and biofouling limitations, particularly in long-duration or high-frequency missions. Nevertheless, future advancements in battery technology and improvement in sensor performance could see AUV-based carbon observations meet the requirements of the observing community while increasing data resolution and reducing the carbon footprint. By leveraging autonomous technology to observe and constrain the marine carbonate system, today’s scientists can better study critical climate issues while ensuring not to contribute to the very problem we seek to understand and mitigate.

## Data Availability

All sensor and CTD data are available from The University of Southampton Data Repository https://doi.org/10.5258/SOTON/D3436.

## Supporting Information Available

Simplified diagram visualizing the carbonate calculation interpolation method; sensor and benchtop determination uncertainties used to derive combined uncertainties; additional data on water column temperature, salinity, dissolved oxygen, density, and fluorescence measured by CTD and ALR during the ST and DT deployments; relationship between pH and dissolved oxygen deployment-wide; and relationships between depth, time, and density versus pH residuals in the ST and DT (PDF)

## References

[ref1] Friedlingstein P.; O’Sullivan M.; Jones M. W.; Andrew R. M.; Bakker D. C. E.; Hauck J.; Landschützer P.; Quéré C. L.; Luijkx I. T.; Peters G. P.; Peters W.; Pongratz J.; Schwingshackl C.; Sitch S.; Canadell J. G.; Ciais P.; Jackson R. B.; Alin S. R.; Anthoni P.; Barbero L.; Bates N. R.; Becker M.; Bellouin N.; Decharme B.; Bopp L.; Brasika I. B. M.; Cadule P.; Chamberlain M. A.; Chandra N.; Chau T.-T.-T.; Chevallier F.; Chini L. P.; Cronin M.; Dou X.; Enyo K.; Evans W.; Falk S.; Feely R. A.; Feng L.; Ford D. J.; Gasser T.; Ghattas J.; Gkritzalis T.; Grassi G.; Gregor L.; Gruber N.; Gürses; Harris I.; Hefner M.; Heinke J.; Houghton R. A.; Hurtt G. C.; Iida Y.; Ilyina T.; Jacobson A. R.; Jain A.; Jarníková T.; Jersild A.; Jiang F.; Jin Z.; Joos F.; Kato E.; Keeling R. F.; Kennedy D.; Goldewijk K. K.; Knauer J.; Korsbakken J. I.; Körtzinger A.; Lan X.; Lefèvre N.; Li H.; Liu J.; Liu Z.; Ma L.; Marland G.; Mayot N.; McGuire P. C.; McKinley G. A.; Meyer G.; Morgan E. J.; Munro D. R.; Nakaoka S.-I.; Niwa Y.; O’Brien K. M.; Olsen A.; Omar A. M.; Ono T.; Paulsen M.; Pierrot D.; Pocock K.; Poulter B.; Powis C. M.; Rehder G.; Resplandy L.; Robertson E.; Rödenbeck C.; Rosan T. M.; Schwinger J.; Séférian R.; Smallman T. L.; Smith S. M.; Sospedra-Alfonso R.; Sun Q.; Sutton A. J.; Sweeney C.; Takao S.; Tans P. P.; Tian H.; Tilbrook B.; Tsujino H.; Tubiello F.; van der Werf G. R.; van Ooijen E.; Wanninkhof R.; Watanabe M.; Wimart-Rousseau C.; Yang D.; Yang X.; Yuan W.; Yue X.; Zaehle S.; Zeng J.; Zheng B. Global Carbon Budget 2023. Earth Syst. Sci. Data 2023, 15, 5301–5369. 10.5194/essd-15-5301-2023.

[ref2] Quéré C. L.; Raupach M. R.; Canadell J. G.; Marland G.; Bopp L.; Ciais P.; Conway T. J.; Doney S. C.; Feely R. A.; Foster P.; Friedlingstein P.; Gurney K.; Houghton R. A.; House J. I.; Huntingford C.; Levy P. E.; Lomas M. R.; Majkut J.; Metzl N.; Ometto J. P.; Peters G. P.; Prentice I. C.; Randerson J. T.; Running S. W.; Sarmiento J. L.; Schuster U.; Sitch S.; Takahashi T.; Viovy N.; van der Werf G. R.; Woodward F. I. Trends in the sources and sinks of carbon dioxide. Nat. Geosci. 2009, 2, 831–836. 10.1038/ngeo689.

[ref3] Barker S.; Ridgwell A. Ocean Acidifcation. Nature Education Knowledge 2012, 3.

[ref4] Caldeira K.; Wickett M. E. Anthropogenic carbon and ocean pH. Nature 2003, 425, 365 10.1038/425365a. 14508477

[ref5] Wolf-Gladrow D. A.; Zeebe R. E.; Klaas C.; Körtzinger A.; Dickson A. G. Total alkalinity: The explicit conservative expression and its application to biogeochemical processes. Marine Chemistry 2007, 106, 287–300. 10.1016/j.marchem.2007.01.006.

[ref6] Zeebe R. E. History of Seawater Carbonate Chemistry, Atmospheric CO2, and Ocean Acidification. Annu. Rev. Earth Planetary Sci. 2012, 40, 141–165. 10.1146/annurev-earth-042711-105521.

[ref7] Feely R. A.; Sabine C. L.; Lee K.; Berelson W.; Kleypas J.; Fabry V. J.; Millero F. J. Impact of anthropogenic CO2 on the CaCO3 system in the oceans. Science 2004, 305, 362–366. 10.1126/science.1097329. 15256664

[ref8] Tans P. An Accounting of the Observed Increase in Oceanic and Atmospheric CO2 and the Outlook for the Future. undefined 2009, 22, 26–35. 10.5670/oceanog.2009.94.

[ref9] Jiang L.-Q.; Carter B. R.; Feely R. A.; Lauvset S. K.; Olsen A. Surface ocean pH and buffer capacity: past, present and future. Sci. Rep. 2019, 9, 18624 10.1038/s41598-019-55039-4. 31819102 PMC6901524

[ref10] Whitt C.; Pearlman J.; Polagye B.; Caimi F.; Muller-Karger F.; Copping A.; Spence H.; Madhusudhana S.; Kirkwood W.; Grosjean L.; Fiaz B. M.; Singh S.; Singh S.; Manalang D.; Gupta A. S.; Maguer A.; Buck J. J.; Marouchos A.; Atmanand M. A.; Venkatesan R.; Narayanaswamy V.; Testor P.; Douglas E.; de Halleux S.; Khalsa S. J. Future Vision for Autonomous Ocean Observations. Front. Mar. Sci. 2020, 7, 697 10.3389/fmars.2020.00697.

[ref11] Byrne R. H. Measuring ocean acidification: New technology for a new era of ocean chemistry. Environ. Sci. Technol. 2014, 48, 5352–5360. 10.1021/es405819p. 24708247

[ref12] Tanhua T.; McCurdy A.; Fischer A.; Appeltans W.; Bax N.; Currie K.; Deyoung B.; Dunn D.; Heslop E.; Glover L. K.; Gunn J.; Hill K.; Ishii M.; Legler D.; Lindstrom E.; Miloslavich P.; Moltmann T.; Nolan G.; Palacz A.; Simmons S.; Sloyan B.; Smith L. M.; Smith N.; Telszewski M.; Visbeck M.; Wilkin J. What we have learned from the framework for ocean observing: Evolution of the global ocean observing system. Front. Mar. Sci. 2019, 6, 436048 10.3389/fmars.2019.00471.

[ref13] Friedlingstein P.; Jones M. W.; O’sullivan M.; Andrew R. M.; Bakker D. C. E.; Hauck J.; Quéré C. L.; Peters G. P.; Peters W.; Pongratz J.; Sitch S.; Canadell J. G.; Ciais P.; Jackson R. B.; Alin S. R.; Anthoni P.; Bates N. R.; Becker M.; Bellouin N.; Bopp L.; Tuyet T.; Chau T.; Chevallier F.; Chini L. P.; Cronin M.; Currie K. I.; Decharme B.; Djeutchouang L. M.; Dou X.; Evans W.; Feely R. A.; Feng L.; Gasser T.; Gilfillan D.; Gkritzalis T.; Grassi G.; Gregor L.; Gruber N.; Gürses Özgür; Harris I.; Houghton R. A.; Hurtt G. C.; Iida Y.; Ilyina T.; Luijkx I. T.; Jain A.; Jones S. D.; Kato E.; Kennedy D.; Goldewijk K. K.; Knauer J.; Korsbakken J. I.; Körtzinger A.; Landschützer P.; Tubiello F.; Werf G. R. V. D.; Vuichard N.; Wada C.; Wanninkhof R.; Watson A. J.; Willis D.; Wiltshire A. J.; Yuan W.; Yue C.; Yue X.; Zaehle S.; Zeng J. Global Carbon Budget 2021. Earth Syst. Sci. Data 2022, 14, 1917–2005. 10.5194/essd-14-1917-2022.

[ref14] Hauck J.; Nissen C.; Landschützer P.; Rödenbeck C.; Bushinsky S.; Olsen A. Sparse observations induce large biases in estimates of the global ocean CO2 sink: an ocean model subsampling experiment. Philos. Trans. R. Soc., A 2023, 381, 20220063 10.1098/rsta.2022.0063. PMC10164466 37150197

[ref15] Lee B. M. C.; DeGrandpre M.; Guthrie J.; Hill V.; Kwok R.; Morison J.; Cox C. J.; Singh H.; Stanton T. P.; Wilkinson J. Emerging technologies and approaches for in situ, autonomous observing in the arctic. Oceanography 2022, 35, 210 10.5670/oceanog.2022.127.

[ref16] Byrne R. Sensors and Systems for in situ Observations of Marine Carbon Dioxide System Variables; Marine Science Faculty Publications: 2010.

[ref17] Millero F. J. The Marine Inorganic Carbon Cycle. Chem. Rev. 2007, 107, 308–341. 10.1021/cr0503557. 17300138

[ref18] Orr J. C.; Epitalon J. M.; Dickson A. G.; Gattuso J. P. Routine uncertainty propagation for the marine carbon dioxide system. Marine Chemistry 2018, 207, 84–107. 10.1016/j.marchem.2018.10.006.

[ref19] Sutton A. J.; Sabine C. L. Emerging applications of longstanding autonomous ocean carbon observations. Oceanography 2023, 36, 148–155. 10.5670/oceanog.2023.209.

[ref20] Carter B. R.; Feely R. A.; Williams N. L.; Dickson A. G.; Fong M. B.; Takeshita Y. Updated methods for global locally interpolated estimation of alkalinity, pH, and nitrate. Limnology and Oceanography: Methods 2018, 16, 119–131. 10.1002/lom3.10232.

[ref21] Juranek L. W.; Feely R. A.; Gilbert D.; Freeland H.; Miller L. A. Real-time estimation of pH and aragonite saturation state from Argo profiling floats: Prospects for an autonomous carbon observing strategy. Geophys. Res. Lett. 2011, 38, 048580 10.1029/2011GL048580.

[ref22] Sabine C.; Sutton A.; McCabe K.; Lawrence-Slavas N.; Alin S.; Feely R.; Jenkins R.; Maenner S.; Meinig C.; Thomas J.; Ooijen E. V.; Passmore A.; Tilbrook B. Evaluation of a New Carbon Dioxide System for Autonomous Surface Vehicles. undefined 2020, 37, 1305–1317. 10.1175/JTECH-D-20-0010.1.

[ref23] Sutton A. J.; Williams N. L.; Tilbrook B. Constraining Southern Ocean CO2 Flux Uncertainty Using Uncrewed Surface Vehicle Observations. Geophys. Res. Lett. 2021, 48, e2020GL091748 10.1029/2020GL091748.

[ref24] Possenti L.; Humphreys M. P.; Bakker D. C. E.; Cobas-García M.; Fernand L.; Lee G. A.; Pallottino F.; Loucaides S.; Mowlem M. C.; Kaiser J. Air-Sea Gas Fluxes and Remineralization From a Novel Combination of pH and O2 Sensors on a Glider. Front. Mar. Sci. 2021, 8, 696772 10.3389/fmars.2021.696772.

[ref25] Roper D.; Harris C. A.; Salavasidis G.; Pebody M.; Templeton R.; Prampart T.; Kingsland M.; Morrison R.; Furlong M.; Phillips A. B.; McPhail S. Autosub Long Range 6000: A Multiple-Month Endurance AUV for Deep-Ocean Monitoring and Survey. IEEE Journal of Oceanic Engineering 2021, 46, 1179–1191. 10.1109/JOE.2021.3058416.

[ref26] Phillips A. B.; Templeton R.; Roper D.; Morrison R.; Pebody M.; Bagley P. M.; Marlow R.; Chaney E.; Burris J.; Consensi A.; Fenucci D.; Fanelli F.; Martin A.; Salavasidis G.; Jones O.; Morris A.; Harris C. A.; Lorenzo A.; Furlong M. Autosub Long Range 1500: A continuous 2000 km field trial. Ocean Engineering 2023, 280, 114626 10.1016/j.oceaneng.2023.114626.

[ref27] Yin T.; Papadimitriou S.; Rérolle V. M. C.; Arundell M.; Cardwell C. L.; Walk J.; Palmer M. R.; Fowell S. E.; Schaap A.; Mowlem M. C.; Loucaides S. A Novel Lab-on-Chip Spectrophotometric pH Sensor for Autonomous In Situ Seawater Measurements to 6000 m Depth on Stationary and Moving Observing Platforms. Environ. Sci. Technol. 2021, 55, 14968–14978. 10.1021/acs.est.1c03517. 34644501

[ref28] Schaap A.; Papadimitriou S.; Mawji E.; Walk J.; Hammermeister E.; Mowlem M.; Loucaides S. An autonomous sensor for in situ measurements of total alkalinity in the ocean. ACS Sens. 2025, 10, 795 10.1021/acssensors.4c02349. 39938881 PMC11877635

[ref29] Monk S. A. Development Of An Autonomous Dissolved Inorganic Carbon Sensor For Oceanic Measurements; University of Southampton: 2020.

[ref30] Cryer S. E.; Evans C.; Fowell S. E.; Andrews G.; Brown P.; Carvalho F.; Degallerie D.; Ludgate J.; Rosado S.; Sanders R.; Strong J. A.; Theophille D.; Young A.; Loucaides S. Characterizing Reef Net Metabolism Via the Diel Co-Variation of pH and Dissolved Oxygen From High Resolution in Situ Sensors. Glob. Biogeochem. Cycles 2023, 37, e2022GB007577 10.1029/2022GB007577.

[ref31] Monk S. A.; Schaap A.; Hanz R.; Borisov S. M.; Loucaides S.; Arundell M.; Papadimitriou S.; Walk J.; Tong D.; Wyatt J.; Mowlem M. Detecting and mapping a CO2 plume with novel autonomous pH sensors on an underwater vehicle. International Journal of Greenhouse Gas Control 2021, 112, 103477 10.1016/j.ijggc.2021.103477.

[ref32] Schaap A.; Koopmans D.; Holtappels M.; Dewar M.; Arundell M.; Papadimitriou S.; Hanz R.; Monk S.; Mowlem M.; Loucaides S. Quantification of a subsea CO2 release with lab-on-chip sensors measuring benthic gradients. International Journal of Greenhouse Gas Control 2021, 110, 103427 10.1016/j.ijggc.2021.103427.

[ref33] Culberson C.; Pytkowicz R. M.; Hawley J. E. Seawater Alkalinity Determination by the pH Method. J. Mar. Res. 1970, 28.

[ref34] Hall P. J.; Aller R. C. Rapid, small-volume, flow injection analysis for SCO2, and NH4+ in marine and freshwaters. Limnology and Oceanography 1992, 37, 1113–1119. 10.4319/lo.1992.37.5.1113.

[ref35] Sayles F. L.; Eck C. An autonomous instrument for time series analysis of TCO2 from oceanographic moorings. Deep-Sea Research Part I: Oceanographic Research Papers 2009, 56, 1590–1603. 10.1016/j.dsr.2009.04.006.

[ref36] Dickson A.; Dickson A. G. The carbon dioxide system in seawater: Equilibrium chemistry and measurements Estimation of uncertainty of seawater pH measurements View project Acidification in Eastern Pacific View project The carbon dioxide system in seawater: equilibrium chemistry and measurements 1 Introduction 1.1, 2010. https://www.researchgate.net/publication/284774361.

[ref37] Sharp J. D.; Byrne R. H. Technical note: Excess alkalinity in carbonate system reference materials. Marine Chemistry 2021, 233, 103965 10.1016/j.marchem.2021.103965.

[ref38] Dickson A.; Chris S.; Christian J. R. Guide to Best Practices for Ocean CO2Measurements; North Pacific Marine Science Organization: 2007; Vol. 3.

[ref39] O’Sullivan D.; Millero F. J. Continual measurement of the total inorganic carbon in surface seawater. Marine Chemistry 1998, 60, 75–83. 10.1016/S0304-4203(97)00079-0.

[ref40] Goyet C.; Snover A. K. High-accuracy measurements of total dissolved inorganic carbon in the ocean: comparison of alternate detection methods. Marine Chemistry 1993, 44, 235–242. 10.1016/0304-4203(93)90205-3.

[ref41] Call M.; Schulz K. G.; Carvalho M. C.; Santos I. R.; Maher D. T. Technical note: Coupling infrared gas analysis and cavity ring down spectroscopy for autonomous, high-temporal-resolution measurements of DIC and δ^13^C–DIC. Biogeosciences 2017, 14, 1305–1313. 10.5194/bg-14-1305-2017.

[ref42] Metrohm. Metrohm Knowledge Base, 2022. https://www.metrohm.com/en_gb/products/titration/ti-touch.html.

[ref43] Becker S.; Aoyama M.; Woodward E. M. S.; Bakker K.; Coverly S.; Mahaffey C.; Tanhua T. GO-SHIP Repeat Hydrography Nutrient Manual: The Precise and Accurate Determination of Dissolved Inorganic Nutrients in Seawater, Using Continuous Flow Analysis Methods. Front. Mar. Sci. 2020, 7, 581790 10.3389/fmars.2020.581790.

[ref44] Birchill A.; Clinton-Bailey G.; Hanz R.; Mawji E.; Cariou T.; White C.; Ussher S.; Worsfold P.; Achterberg E.; Mowlem M. Realistic measurement uncertainties for marine macronutrient measurements conducted using gas segmented flow and Lab-on-Chip techniques. Talanta 2019, 200, 228–235. 10.1016/j.talanta.2019.03.032. 31036178

[ref45] Winkler L. W. Die Bestimmung des im Wasser gelösten Sauerstoffes. Berichte der deutschen chemischen Gesellschaft 1888, 21, 2843–2854. 10.1002/cber.188802102122.

[ref46] Carpenter J. H. THE CHESAPEAKE BAY INSTITUTE TECHNIQUE FOR THE WINKLER DISSOLVED OXYGEN METHOD. Limnology and Oceanography 1965, 10, 141–143. 10.4319/lo.1965.10.1.0141.

[ref47] Langdon C. Determination of Dissolved Oxygen in Seaweater By Winkler Titration using Amperometric Technique. In GO-SHIP Repeat Hydrography Manual: A Collection of Expert Reports and Guidelines. Version 1; ICPO: 2010; https://repository.oceanbestpractices.org/handle/11329/380.

[ref48] McIntosh P. C. Oceanographic data interpolation: Objective analysis and splines. Journal of Geophysical Research: Oceans 1990, 95, 13529–13541. 10.1029/JC095iC08p13529.

[ref49] Ledoux H.; Gold C. An Efficient Natural Neighbour Interpolation Algorithm for Geoscientific Modelling. Developments in Spatial Data Handling. 2005, 97–108. 10.1007/3-540-26772-7_8.

[ref50] Amidror I. Scattered data interpolation methods for electronic imaging systems: a survey. Journal of Electronic Imaging 2002, 11, 157–176. 10.1117/1.1455013.

[ref51] Sibson R. A brief description of natural neighbour interpolation. In Interpreting multivariate data; John Wiley & Sons: 1981; pp 21–36.

[ref52] Sambridge M.; Braun J.; McQueen H. Geophysical parametrization and interpolation of irregular data using natural neighbours. Geophysical Journal International 1995, 122, 837–857. 10.1111/j.1365-246X.1995.tb06841.x.

[ref53] McDougall T. J.; Barker P. M. Getting started with TEOS-10 and the Gibbs Seawater (GSW) Oceanographic Toolbox. 2011; 28pp, SCOR/IAPSO WG127, ISBN 978-0-646-55621-5.

[ref54] Gomis D.; Pedder M. A.; Viúdez A. Recovering spatial features in the ocean: performance of isopycnal vs. isobaric analysis. Journal of Marine Systems 1997, 13, 205–224. 10.1016/S0924-7963(96)00116-9.

[ref55] Lozier M. S.; McCartney M. S.; Owens W. B. Anomalous Anomalies in Averaged Hydrographic Data. Journal of Physical Oceanography 1994, 24, 2624–2638. 10.1175/1520-0485(1994)024<2624:AAIAHD>2.0.CO;2.

[ref56] Ridgway K. R.; Dunn J. R.; Wilkin J. L. Ocean Interpolation by Four-Dimensional Weighted Least Squares—Application to the Waters around Australasia. Journal of Atmospheric and Oceanic Technology 2002, 19, 1357–1375. 10.1175/1520-0426(2002)019<1357:OIBFDW>2.0.CO;2.

[ref57] Monterey G. I.; Levitus S. Seasonal variability of mixed layer depth for the world ocean; NOAA atlas NESDIS; NOAA: 1997; Vol. 14.

[ref58] Levitus S. Climatological atlas of the world ocean; US Department of Commerce, National Oceanic and Atmospheric Administration: 1982; Vol. 13.

[ref59] de Boyer Montégut C.; Madec G.; Fischer A. S.; Lazar A.; Iudicone D. Mixed layer depth over the global ocean: An examination of profile data and a profile-based climatology. J. Geophys. Res.: Oceans 2004, 109, 02378 10.1029/2004JC002378.

[ref60] Lewis E. R.; Wallace D. W. R.; Allison L. J. Program Developed for CO2 System Calculations; Oak Ridge National Lab.: 1998.

[ref61] Sharp J. D.; Pierrot D.; Humphreys M. P.; Epitalon J.-M.; Orr J. C.; Lewis E. R.; Wallace D. W. CO2SYSv3 for MATLAB; Zendo: 2023.

[ref62] van Heuven S.; Pierrot D.; Rae J.; Lewis E.; Wallace D. W. R. CO2SYS v 1.1, MATLAB program developed for CO2 system calculations; CDIAC: 2011.

[ref63] Lueker T. J.; Dickson A. G.; Keeling C. D. Ocean pCO2 calculated from dissolved inorganic carbon, alkalinity, and equations for K1 and K2: validation based on laboratory measurements of CO2 in gas and seawater at equilibrium. Marine Chemistry 2000, 70, 105–119. 10.1016/S0304-4203(00)00022-0.

[ref64] Dickson A. G. Standard potential of the reaction: AgCl(s) + 12H2(g) = Ag(s) + HCl(aq), and and the standard acidity constant of the ion HSO4- in synthetic sea water from 273.15 to 318.15 K. J. Chem. Thermodyn. 1990, 22, 113–127. 10.1016/0021-9614(90)90074-Z.

[ref65] Perez F. F.; Fraga F. Association constant of fluoride and hydrogen ions in seawater. Marine Chemistry 1987, 21, 161–168. 10.1016/0304-4203(87)90036-3.

[ref66] Lee K.; Kim T.-W.; Byrne R. H.; Millero F. J.; Feely R. A.; Liu Y.-M. The universal ratio of boron to chlorinity for the North Pacific and North Atlantic oceans. Geochim. Cosmochim. Acta 2010, 74, 1801–1811. 10.1016/j.gca.2009.12.027.

[ref67] JCGM; BIPM; IEC; IFCC; ILAC; ISO; IUPAC; IUPAP; OIMIL. Evaluation of measurement data-Guide to the expression of uncertainty in measurement. In JCGM 100:2008; JCGM: 2008.

[ref68] Kitidis V.; Hardman-Mountford N. J.; Litt E.; Brown I.; Cummings D.; Hartman S.; Hydes D.; Fishwick J. R.; Harris C.; Martinez-Vicente V.; Malcolm E.; Woodward S.; Smyth T. J. Seasonal dynamics of the carbonate system in the Western English Channel. Cont. Shelf Res. 2012, 42, 30–40. 10.1016/j.csr.2012.04.012.

[ref69] Marrec P.; Cariou T.; Collin E.; Durand A.; Latimier M.; Macé E.; Morin P.; Raimund S.; Vernet M.; Bozec Y. Seasonal and latitudinal variability of the CO2 system in the western English Channel based on Voluntary Observing Ship (VOS) measurements. Marine Chemistry 2013, 155, 29–41. 10.1016/j.marchem.2013.05.014.

[ref70] Nehir M.; Esposito M.; Loucaides S.; Achterberg E. P. Field Application of Automated Spectrophotometric Analyzer for High-Resolution In Situ Monitoring of pH in Dynamic Estuarine and Coastal Waters. Front. Mar. Sci. 2022, 9, 891876 10.3389/fmars.2022.891876.

[ref71] Hemming M. P.; Kaiser J.; Heywood K. J.; Bakker D. C. E.; Boutin J.; Shitashima K.; Lee G.; Legge O.; Onken R. Measuring pH variability using an experimental sensor on an underwater glider. Ocean Sci. 2017, 13, 427–442. 10.5194/os-13-427-2017.

[ref72] Saba G. K.; Wright-Fairbanks E.; Chen B.; Cai W.-J.; Barnard A. H.; Jones C. P.; Branham C. W.; Wang K.; Miles T. The Development and Validation of a Profiling Glider Deep ISFET-Based pH Sensor for High Resolution Observations of Coastal and Ocean Acidification. Front. Mar. Sci. 2019, 6, 664 10.3389/fmars.2019.00664.

[ref73] Johnson K. S.; Jannasch H. W.; Coletti L. J.; Elrod V. A.; Martz T. R.; Takeshita Y.; Carlson R. J.; Connery J. G. Deep-Sea DuraFET: A Pressure Tolerant pH Sensor Designed for Global Sensor Networks. Anal. Chem. 2016, 88, 3249–3256. 10.1021/acs.analchem.5b04653. 26890717

[ref74] Takeshita Y.; Jones B. D.; Johnson K. S.; Chavez F. P.; Rudnick D. L.; Blum M.; Conner K.; Jensen S.; Long J. S.; Maughan T.; Mertz K. L.; Sherman J. T.; Warren J. K. Accurate pH and O2Measurements from Spray Underwater Gliders. Journal of Atmospheric and Oceanic Technology 2021, 38, 181–195. 10.1175/JTECH-D-20-0095.1.

[ref75] Rérolle V.; Ruiz-Pino D.; Rafizadeh M.; Loucaides S.; Papadimitriou S.; Mowlem M.; Chen J. Measuring pH in the Arctic Ocean: Colorimetric method or SeaFET?. Methods in Oceanography 2016, 17, 32–49. 10.1016/j.mio.2016.05.006.

[ref76] Hartman S. E.; Humphreys M. P.; Kivimäe C.; Woodward E. M. S.; Kitidis V.; Mcgrath T.; Hydes D. J.; Greenwood N.; Hull T.; Ostle C.; Pearce D. J.; Sivyer D.; Stewart B. M.; Walsham P.; Painter S. C.; Mcgovern E.; Harris C.; Griffiths A.; Smilenova A.; Clarke J.; Davis C.; Sanders R.; Nightingale P. Seasonality and spatial heterogeneity of the surface ocean carbonate system in the northwest European continental shelf. Prog. Oceanogr. 2014, 177, 101909 10.1016/j.pocean.2018.02.005.

[ref77] Sonnichsen C.; Atamanchuk D.; Hendricks A.; Morgan S.; Smith J.; Grundke I.; Luy E.; Sieben V. J. An Automated Microfluidic Analyzer for In Situ Monitoring of Total Alkalinity. ACS Sensors 2023, 8, 344–352. 10.1021/acssensors.2c02343. 36602412 PMC9888396

[ref78] Qiu L.; Li Q.; Yuan D.; Chen J.; Xie J.; Jiang K.; Guo L.; Zhong G.; Yang B.; Achterberg E. P. High-Precision In Situ Total Alkalinity Analyzer Capable of Month-Long Observations in Seawaters. ACS Sensors 2023, 8, 2702–2712. 10.1021/acssensors.3c00552. 37357408

[ref79] Spaulding R. S.; DeGrandpre M. D.; Beck J. C.; Hart R. D.; Peterson B.; Carlo E. H. D.; Drupp P. S.; Hammar T. R. Autonomous in Situ Measurements of Seawater Alkalinity. Environ. Sci. Technol. 2014, 48, 9573–9581. 10.1021/es501615x. 25051401

[ref80] Cai W.-J.; Hu X.; Huang W.-J.; Jiang L.-Q.; Wang Y.; Peng T.-H.; Zhang X. Alkalinity distribution in the western North Atlantic Ocean margins. J. Geophys. Res.: Oceans 2010, 115, C08014 10.1029/2009JC005482.

[ref81] Cronin M. F.; Anderson N. D.; Zhang D.; Berk P.; Wills S. M.; Serra Y.; Kohlman C.; Sutton A. J.; Honda M. C.; Kawai Y.; Yang J.; Thomson J.; Lawrence-Slavas N.; Eyre J. R.; Meinig C. PMEL ocean climate stations as reference time series and research aggregate devices. Oceanography 2023, 36, 46–53. 10.5670/oceanog.2023.224.

[ref82] Clarke J. S.; Achterberg E. P.; Connelly D. P.; Schuster U.; Mowlem M. Developments in marine pCO2 measurement technology; towards sustained in situ observations. TrAC Trends in Analytical Chemistry 2017, 88, 53–61. 10.1016/j.trac.2016.12.008.

[ref83] Newton J.; Jewett E.; Tilbrook B.; Bellerby R.; Chai F.; Chen C.-T. A.; Dupont S.; Feely R. A.; Findlay H. S.; Hansson L.; Hassoun A.; Isensee K.; Khokiattiwong S.; Mayorga E.; McIntosh R.; Pfeil B.; Schoo K. L.; Shaltout N.; Vargas C. Global Ocean Acidification Observing Network (GOA-ON) Implementation Strategy; GOA-ON: 2019; p 2019.

[ref84] Sloyan B. M.; Wanninkhof R.; Kramp M.; Johnson G. C.; Talley L. D.; Tanhua T.; McDonagh E.; Cusack C.; O’Rourke E.; McGovern E.; Katsumata K.; Diggs S.; Hummon J.; Ishii M.; Azetsu-Scott K.; Boss E.; Ansorge I.; Perez F. F.; Mercier H.; Williams M. J. M.; Anderson L.; Lee J. H.; Murata A.; Kouketsu S.; Jeansson E.; Hoppema M.; Campos E. The Global Ocean Ship-Based Hydrographic Investigations Program (GO-SHIP): A Platform for Integrated Multidisciplinary Ocean Science. Front. Mar. Sci. 2019, 6, 445 10.3389/fmars.2019.00445.

