# Supplementary material for: New Capability in Autonomous Ocean Carbon Observations Using the Autosub Long-Range AUV Equipped with Novel pH and Total Alkalinity Sensors
Source: Environ Sci Technol. 2025 Apr 1;59(14):7129–44. doi: 10.1021/acs.est.4c10139 (PMC12004916; doi:10.1021/acs.est.4c10139)
Supplement: Supplementary file 1 — es4c10139_si_001.pdf [file es4c10139_si_001.pdf]

Supporting Information for:

“New capability in autonomous ocean carbon observations using the Autosub Long-Range AUV equipped with novel pH and Total Alkalinity sensors”

Emily M. Hammermeister[1,2], Stathys Papadimitriou[2], Martin Arundell[2], Jake Ludgate[2], Allison Schaap[2], Matthew C. Mowlem[2], Sara E. Fowell[2], Edward Chaney[2], and Socratis Loucaides[2]*

1. School of Ocean and Earth Sciences, University of Southampton, SO17 1BJ, Southampton, United Kingdom

2. National Oceanography Centre, European Way, SO14 3ZH, Southampton, United Kingdom

*Corresponding author email: e.m.hammermeister@soton.ac.uk

Supporting Information summary: 6 pages, 6 figures, 1 table.

Interpolation simplified diagram

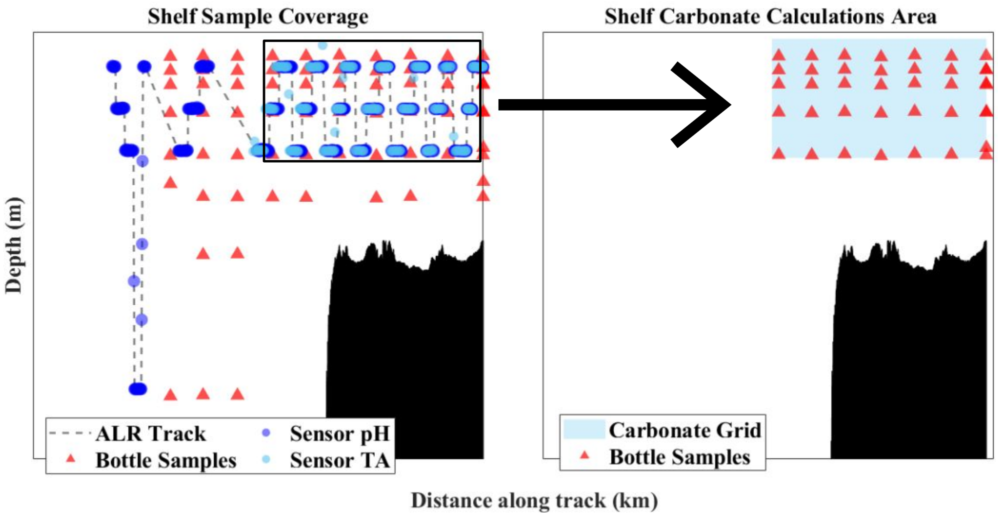

Figure SI.1. Visual representation of 'standardised' grid of $pH_{T\text{-}ALR}$ and TA_{ALR} interpolated data coverage to be used for CO2SYS computation.

Input Parameter	Sensors onboard ALR	Discrete Bottled Samples
uTA (μmolkg^{-1})	7	3
upH (pH units)	0.010	0.005
uDIC (μmolkg^{-1})	n/a	6
uTemperature (°C)	0.002	0.001
uSalinity f	0.003	0.003

Table SI.1. Uncertainty values for each input parameter used for error propagation calculations using CO2SYS *errors.m* routine and standard combined uncertainty calculations using Equation 3 (main manuscript). Uncertainties for parameters determined from technology manufacturers specifications as outlined in main text.

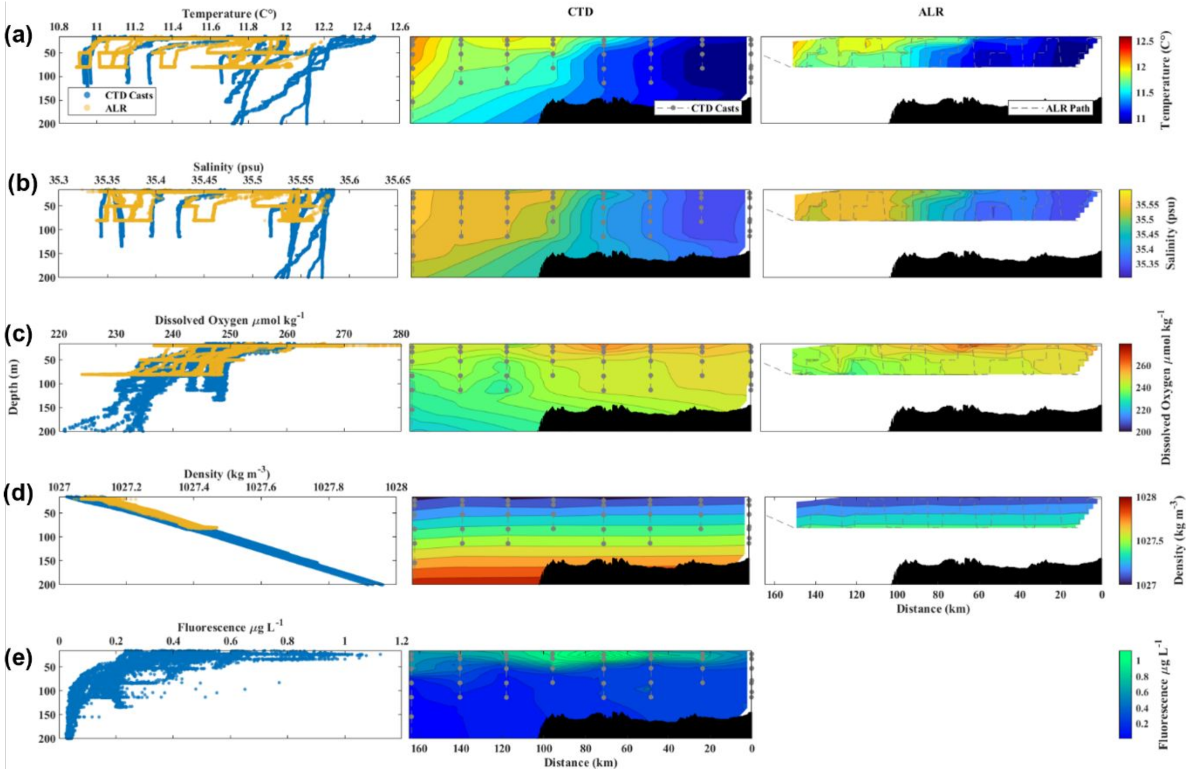

SI. Figure 2. Comparative profiles and spatial contour maps of parameters measured by CTD casts and ALR across the Shelf transect. Each row corresponds to a different parameter: (a) Temperature (°C) profiles with depth. The left panel shows CTD casts (blue) and ALR data (orange). The middle panel shows a contour plot of the CTD data, and the right panel shows a contour plot of the ALR data. Contour lines and their colour are defined by the parameter shown with depth along distance of Shelf Transect. The grey circles in the middle plots indicate CTD bottle locations and while the dashed grey lines in the right plots represent the ALR path. (b) Salinity (PSU) profiles and contours with the same format as in (a). (c) Dissolved oxygen (μmol/kg) profiles and contours with the same format as in (a). (d) Density (kg/m³) profiles with the same format as in (a). (e) Fluorescence (μg/L) profiles with the same format as in (a). There was no fluorometer onboard the ALR which is why there is no depth profile or contour map in row (e) with ALR data. The ALR contour maps in the third column of rows a-d do not go to the same depth as the CTD contour maps in the second column because the ALR's maximum depth measuring these parameters in the shelf transect was 80m.

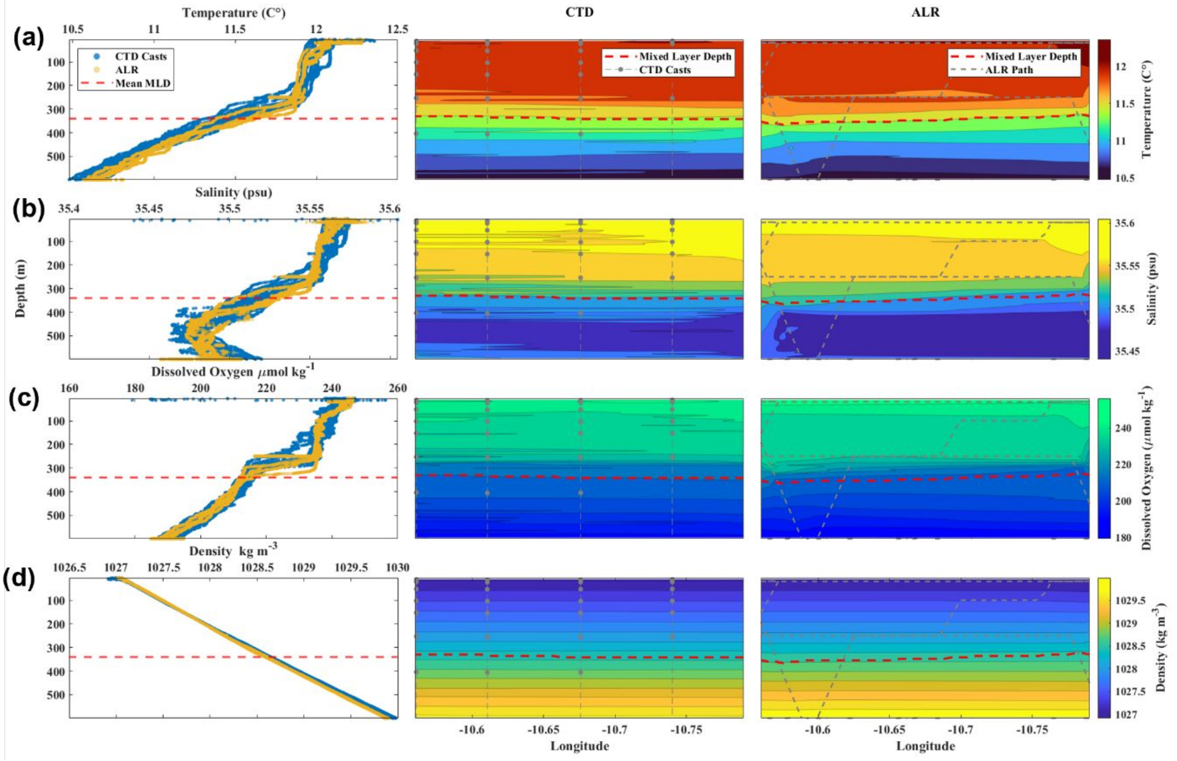

SI. Figure 3. Comparative profiles and spatial contour maps of parameters measured by CTD casts and ALR across the Deep Transect. The depth profiles on the leftmost plots for all rows a-d show CTD casts (blue) and ALR data (orange) of respective parameters plotted against depth. The red dashed line in all subplots represent the mean Mixed Layer Depth (MLD). The grey circles in the middle plots indicate CTD bottle locations and while the dashed grey lines in the right plots represent the ALR path. Contour lines and their colour are defined by the parameter shown with depth along longitude transect. Each row corresponds to a different parameter: (a) Temperature (°C) profiles with depth. The middle panel shows a contour plot of the CTD data, and the right panel shows a contour plot of the ALR data. (b) Salinity (PSU) depth profiles and contour maps with the same format as in (a). (c) Dissolved oxygen (μmol/kg) depth profiles and contour maps with the same format as in (a). (d) Density (kg/m³) depth profiles and contour maps with the same format as in (a).

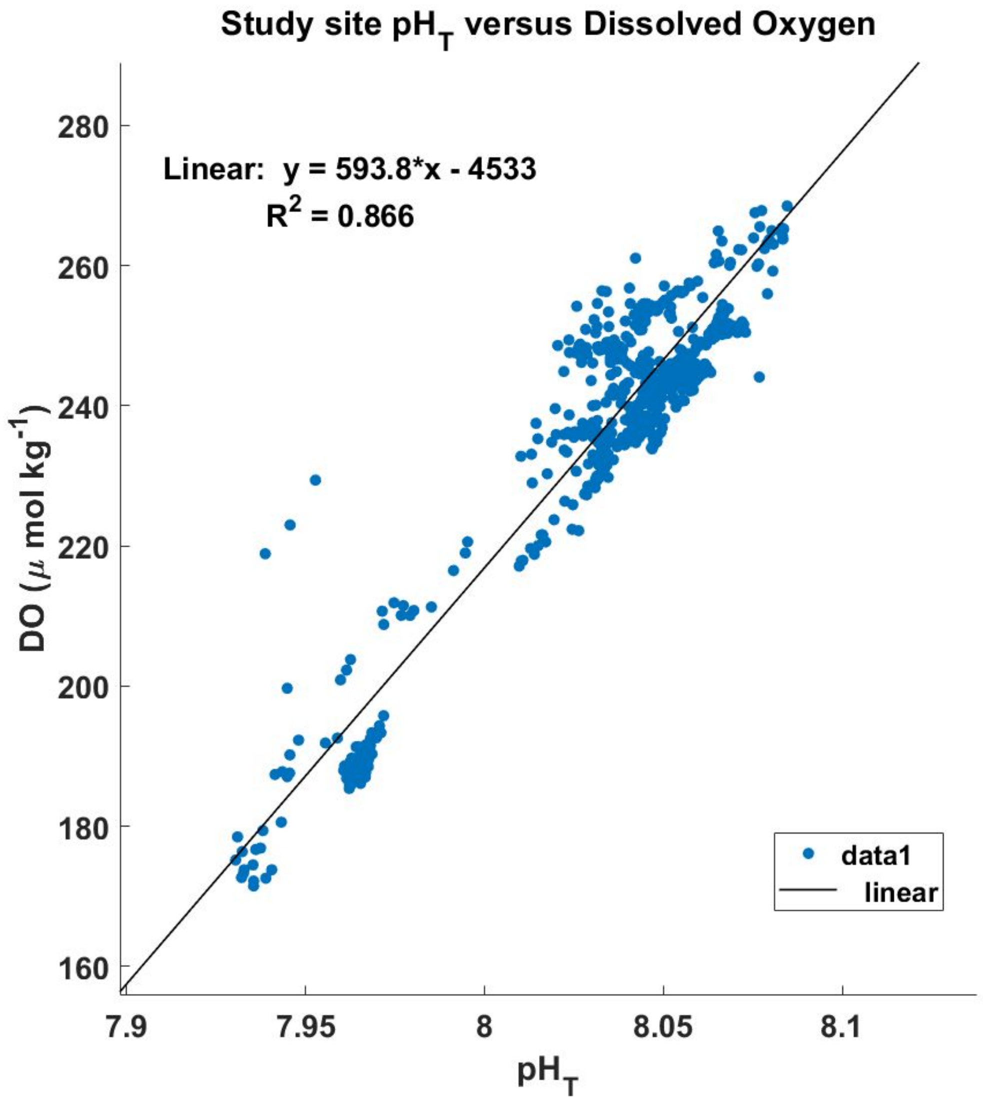

SI. Figure 4. Plot shows all CTD and ALR from both ST and DT measured data of pH_T (x axis) plotted against Dissolved Oxygen (DO) (y axis) to reveal a strong positive linear relationship where $R^2 = 0.866$, p-value < 0.05, and n = 950.

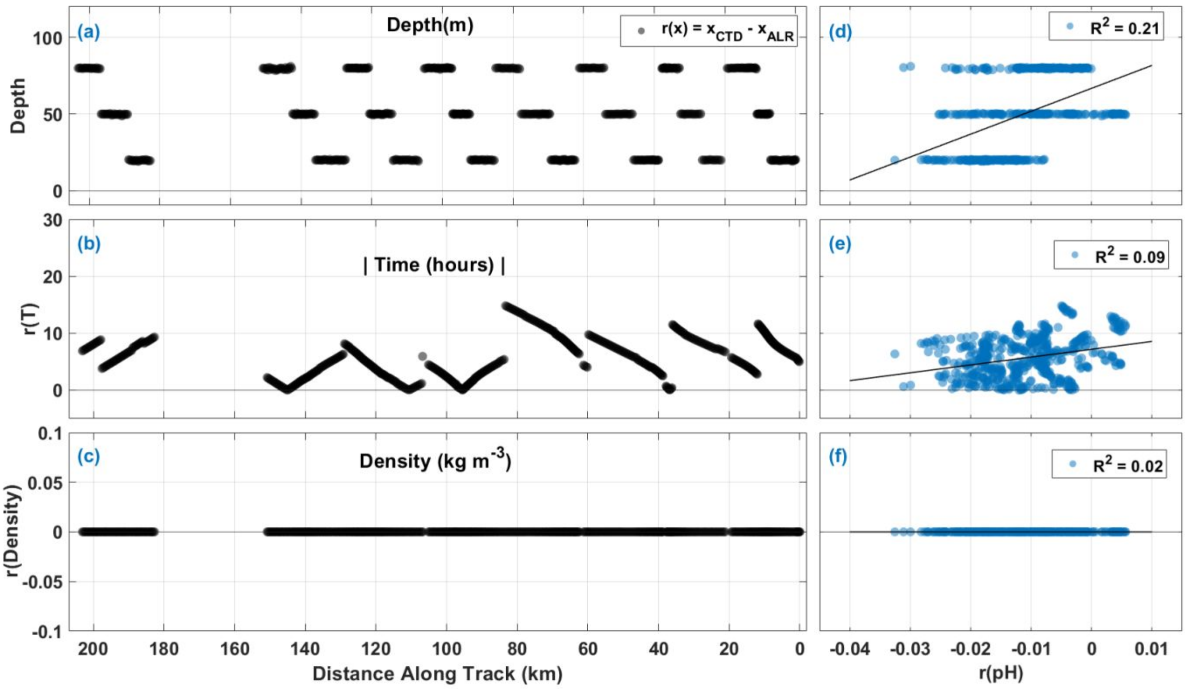

SI. Figure 5. Comparison of depth (a,d), time between CTD and ALR measurements r(Time) (b,e), and density residuals r(Density) (c,f) with respect to transect distance (x-axis in a-c) and pH_T residuals (x-axis in d-f) in the Shelf Transect.

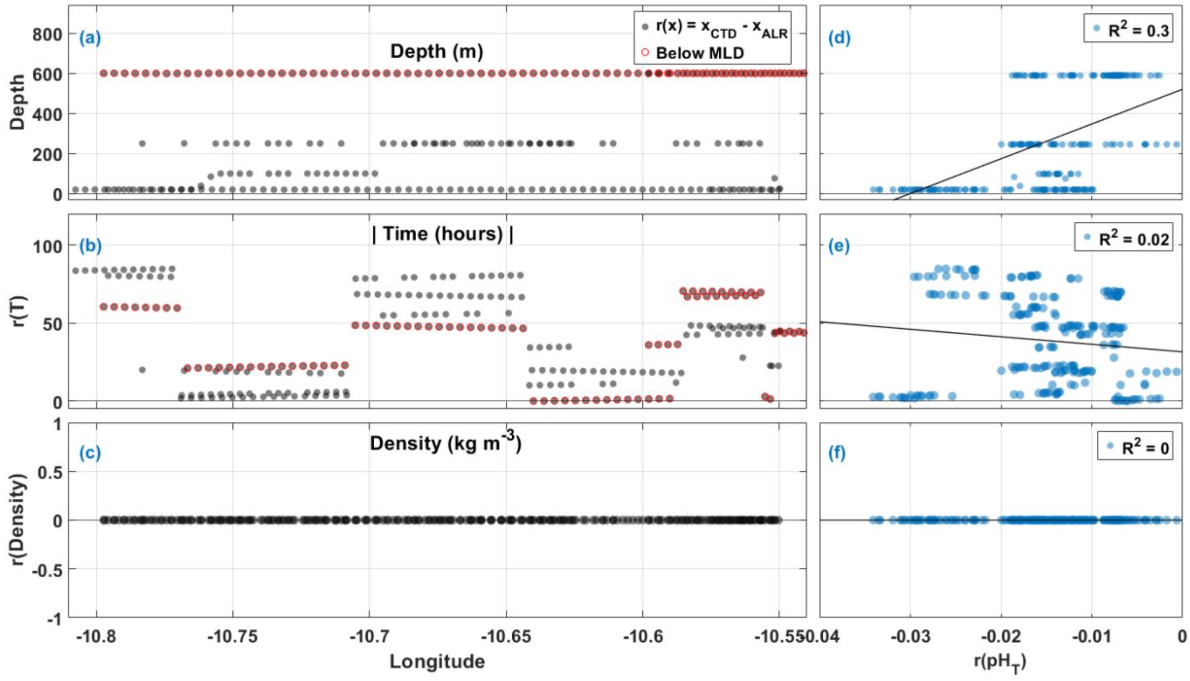

SI. Figure 6. Comparison of depth (a,d), time between CTD and ALR measurements r(Time) (b,e), and density residuals r(Density) (c,f) with respect to longitude (x-axis in a-c) and pH_T residuals (x-axis in d-f) in the Deep Transect. In (a,b) residuals below the MLD (< 345m) are outlined in red.
